# Mechanobiology-based strategies for the maturation of biofabricated cartilage constructs

**DOI:** 10.3389/fbioe.2026.1717769

**Published:** 2026-06-04

**Authors:** Iván López-González, Noelia Campillo, José Manuel Baena

**Affiliations:** 1 REGEMAT – Bioengineering and Advanced Therapies (BAT) Institute – CEU UCH Corporate Chair, Universidad Cardenal Herrera-CEU, CEU Universities, Valencia, Spain; 2 Biofabrication and Cell Culture Laboratory, REGEMAT 3D S.L., Granada, Spain; 3 Biofabrication Group, Department of Pharmacy, School of Health Sciences, Universidad Cardenal Herrera-CEU, CEU Universities, Valencia, Spain

**Keywords:** articular cartilage, biofabrication, biomaterials, bioprinting, cartilage tissue engineering, mechanobiology, regenerative medicine

## Abstract

Recent advancements in 3D bioprinting and biofabrication offer promising avenues for tissue engineering (TE) and regenerative medicine (RM), particularly in addressing musculoskeletal conditions such as articular cartilage lesions. Despite significant progress in generating bioartificial tissue substitutes *ex vivo*, achieving functional tissues with accurate histological structure and physiological function remains a considerable challenge. This is largely due to the absence of crucial physiological stimuli *in vitro* that normally govern cell characteristics and functionality in native tissues. Therefore, the application of biochemical, physical, and mechanical stimuli that accurately mimic the *in vivo* environment is essential for the maturation of 3D constructs into functional tissues. This review explores the latest developments in mechanobiology-based strategies, biomaterials, and bioreactor systems that are designed to produce functional cartilage tissue. We delve into various types of stimuli, including mechanical (e.g., compression, tension, shear, and hydrostatic pressure), biochemical (e.g., growth factors and nanoparticles), and electrical stimulation, and their profound influence on cell differentiation and extracellular matrix (ECM) synthesis. This article highlights the critical role of advanced biomaterials—such as thermoplastics and hydrogels (natural and synthetic, including collagen, gelatin, alginate, and nanocellulose)—in providing structural support and mimicking native tissue properties. Furthermore, we discuss the indispensable role of computational simulation methods, such as the finite element method (FEM), artificial neural networks (ANN), and molecular dynamics (MD), in predicting scaffold behavior, optimizing designs, and understanding complex biological interactions within engineered cartilage. Finally, the review examines the current bioreactor technologies, emphasizing the need for automated systems that are capable of precisely replicating the intricate anatomical configuration and physiological load distribution of human joints, as exemplified by innovative approaches such as the BMAP® Knee bioreactor, to accelerate the translation of engineered tissues from laboratory to clinic. This comprehensive overview aims to serve as a valuable resource for researchers navigating the multidisciplinary challenges and opportunities in cartilage tissue engineering and regenerative medicine.

## 1 Introduction

Recent developments in additive manufacturing (AM) and three-dimensional (3D) printing technologies have enabled the fabrication of complex meshed structures combining biomaterials with living cells, thereby facilitating the generation of advanced 3D cultures with improved viability and architectural control. Within this context, 3D bioprinting has emerged as a powerful tool in tissue engineering (TE) and regenerative medicine (RM), expanding the range of possibilities for producing anatomically relevant constructs (Xu et al., 2013; Baena et al., 2019; Alizadeh Sardroud et al., 2023).

Despite these advances, achieving fully functional tissues that recapitulate the histological organization and mechanical behavior of the native counterparts remains a major challenge (Murphy and Atala, 2014). Beyond structural fabrication, it is essential to promote the cellular activities required for extracellular matrix (ECM) deposition and tissue maturation (Strobel et al., 2020). This underscores the importance of research not only on biomaterials with suitable printability and mechanical properties but also on the biological and mechanical stimuli that govern tissue development (Ashammakhi et al., 2019; van Kampen et al., 2023).

Advances in 3D bioprinting hold the potential to revolutionize RM, drug development, and personalized medical interventions aimed at extending healthy life expectancy (HLE). To address these ambitious goals, it is essential to establish multidisciplinary research teams that integrate expertise in biomaterials engineering, mechanobiology, cell culture, and biofabrication technologies. Additionally, artificial intelligence and machine learning approaches for imaging and predictive modeling offer new opportunities to accelerate the development of viable tissues and organs (Verma et al., 2023).

This review does not aim to exhaustively cover all the published work in these areas. Instead, it intends to provide researchers with a structured and accessible overview of the recent developments in bioprinting, mechanobiology, biomaterials, and dynamic culture systems, serving as a foundation to explore multidisciplinary interactions that drive functional tissue generation.

### 1.1 The origin of the problem

Musculoskeletal disorders are among the leading causes of human disability worldwide, with lesions of the osteochondral interface accounting for a significant portion of these disorders (Yildirim et al., 2023). The growing prevalence of these conditions, particularly in the aging population, highlights the urgent need for new regenerative strategies that are capable of restoring functional tissue. The limited regenerative capacity of cartilage is attributed to several factors, including the scarcity of cells with reparative potential, altered mechanical environments, unresolved inflammation, and metabolic dysregulation (Muthu et al., 2023).

The emergence of 3D bioprinting has enabled controlled *ex vivo* fabrication of tissue substitutes with increasing complexity (Oberdiek et al., 2021). However, bioprinted constructs often lack the maturity and histological organization required to perform their biological functions. One of the main limitations is the absence of the physiological stimuli that cells normally experience *in vivo*, which are critical for defining their phenotype, function, and matrix production (Maul et al., 2011). For this reason, applying biochemical, physical, and mechanical stimuli that mimic native cues is essential to promote the maturation of 3D constructs into functional tissues (Selden and Fuller, 2018).

### 1.2 Complexity of the problem

Articular cartilage is a highly specialized avascular tissue that covers the articular surfaces of bones, providing a low-friction, shock-absorbing surface for joints. It is primarily composed of hyaline cartilage, which includes chondrocytes embedded in an ECM rich in type-II collagen and proteoglycans (PGs). This composition provides exceptional mechanical properties but also restricts cell mobility, which contributes to the tissue’s poor regenerative capacity (Zhang et al., 2009). The absence of vascular, neural, and lymphatic networks, along with the scarcity of endogenous progenitor cells, further hinders the repair process. Consequently, restoring full functionality after cartilage injury remains a significant clinical challenge (Krishnan and Grodzinsky, 2018).

Osteoarthritis (OA), a degenerative condition affecting articular cartilage and subchondral bone, is among the most disabling diseases in developed countries. Approximately 10% of men and 18% of women aged over 60 years suffer from symptomatic OA, including the moderate and severe forms (Long et al., 2022). In younger patients, total knee arthroplasty is generally avoided, making regenerative approaches—such as microfracture, autologous chondrocyte implantation (ACI), and osteochondral transplantation—more suitable alternatives (Martin et al., 2017).

### 1.3 Motivation of the publication

Bioprinting technologies offer unique advantages for fabricating customized tissue-engineered constructs with controlled architectures and tunable degradation profiles (Souness et al., 2018; Xie et al., 2020). However, fabrication alone represents only one part of the solution to effectively treat patients. A thorough understanding of the processes governing ECM establishment, maintenance, remodeling, and repair—and the stimuli that regulate these events—is essential.

This review synthesizes the recent advancements, technologies, and research findings related to this multidisciplinary field, emphasizing the interplay between bioprinting, biomaterials, mechanobiology, computational modeling, and bioreactor-based dynamic culture systems.

## 2 Biomaterials for 3D bioprinting in cartilage tissue engineering

Biomaterials used in cartilage TE must provide adequate biocompatibility, controlled biodegradation, and mechanical properties that are compatible with chondrogenesis. In the context of 3D bioprinting, hydrogels and thermoplastics represent the most relevant classes of materials due to their ability to mimic the hydrated microenvironment of cartilage or provide structural support during maturation. Hydrogels serve as cell-laden matrices with tunable viscoelasticity, while thermoplastics enable the fabrication of scaffolds with defined architecture and load-bearing capacity.

For bioprinting applications in cartilage TE, biomaterials must also exhibit suitable printability, viscosity, and shape fidelity to generate constructs with predefined porosity and mechanical performance. Matching the biodegradation rate with matrix deposition is particularly important for articular cartilage, where insufficient scaffold persistence or excessive degradation may compromise tissue integrity. Therefore, the selection and formulation of biomaterials focus on achieving a balance between printability, mechanical robustness, and biological functionality.

Scaffolds should have suitable architecture and strength to serve their intended function and provide a 3D environment that is desirable for the production of tissues (Yeong et al., 2004). Key scaffold requirements include: i) promoting cell viability, differentiation, and biological ECM production; ii) exhibiting controlled and predictable degradation; iii) integrating with the surrounding native tissue; iv) allowing vascularization and the diffusion of nutrients and waste products; v) providing location-specific mechanical integrity (Chung and Burdick, 2008).

**FIGURE 1:**
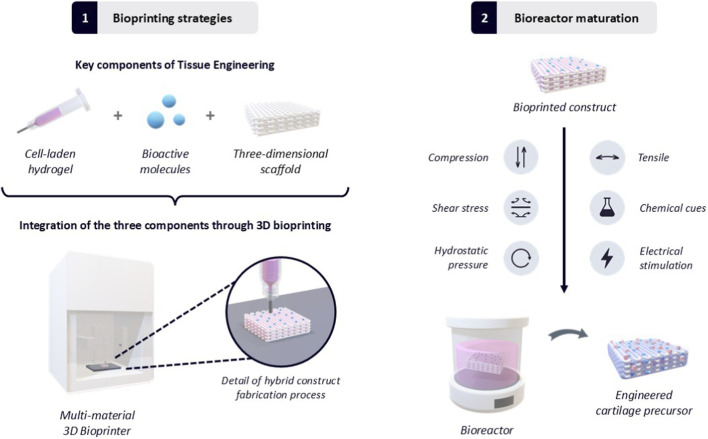
Process from bioprinting to *in vitro* maturation of cartilage-like constructs (López-González, 2025). (1) The process begins with bioprinting strategies that integrate the three key components of tissue engineering—cell-laden hydrogels, bioactive molecules, and three-dimensional scaffolds—through a multi-material 3D bioprinting process. (2) The resultant construct is subsequently cultured in a bioreactor that provides controlled stimuli to support tissue development and functional maturation, ultimately yielding an engineered cartilage-like construct.

Although some components may vary or be absent, three main elements are generally required for successful TE approaches. First, a scaffold to provide adequate 3D structure and spatial support; second, a bioink containing a hydrogel with stem cells (SCs) capable of differentiating and maintaining specific phenotypes; and third, bioactive substances, such as growth factors, cytokines, hormones, or exosomes, to elicit cell differentiation toward a specific lineage (Peng et al., 2023) (Figure 1).

Developing new strategies to produce the cell-laden scaffolds needed for the regeneration of tissue injuries is paramount. In addition to the bioprinting process, this also involves defining new mechanobiology-based procedures to stimulate the resultant construct consisting of the scaffold, cell-laden bioinks, and biomolecules to create a living tissue with adequate structure, composition, and function.

### 2.1 Natural hydrogels

Natural hydrogels are widely used as cell carriers and ECM-mimetic matrices in cartilage bioprinting due to their inherent biocompatibility and high water content. Hydrogels are highly absorbent materials containing over 90% of water. These hydrophilic polymer networks swell to an equilibrium volume while retaining their shape. They can be used to fabricate scaffolds that are suitable for soft tissue (e.g., nerves, blood vessels, and tendons). In addition, they can serve as matrices to cover or fill the inner volumes of another construct or as support networks for embedding cells and other biomolecules. It is possible to produce hydrogels with a significant portion of hydrophobic polymers, sometimes found as colloidal gels, where water is the dispersion medium. Based on their origin and properties, hydrogels can be classified as natural or synthetics; degradable or non-degradable; physical or chemical; neutral, cationic, anionic, or amphiphilic; and amorphous or semi-crystalline, among others (Baroli, 2007). They also possess a degree of flexibility that mimics the properties of native tissues due to their significant water content. One of the main disadvantages of processing hydrogels is the difficulty to shape them into predesigned geometries. Some 3D-printing technologies are available or can be adapted to print such materials. Hydrogels can also be used in hard tissue regeneration as a component attached to the rigid mesh containing cells or other signaling molecules, such as a coating to improve biocompatibility or to deliver growth factors. Rapid prototyping techniques have shown high potential for the production of scaffolds with the purpose of cell seeding and/or cell encapsulation (Billiet et al., 2012).

#### 2.1.1 Alginate

Alginate is a natural polysaccharide derived from seaweed, and it is biodegradable, non-toxic, and non-inflammatory, with tunable porosity and excellent printability (Schuurman et al., 2011). To overcome the limited bioactivity and weak mechanics, alginate is frequently blended with other biologically active hydrogels or mechanically robust biomaterials for cartilage TE (Jia et al., 2014). Specific compositions and optimized printing parameters yield high printing capability, making alginate a practical component for cartilage applications (Gorronogoitia et al., 2022; Greco et al., 2022).

#### 2.1.2 Collagen (type I/II)

Collagen is the most abundant protein of the ECM and has been used in biomedical and cosmetic applications, along with being analyzed for cartilage regeneration (Beketov et al., 2021). It has gathered significant attention for its biocompatibility and ability to mimic native tissue environments. Different types of collagen (type I, II, III, and IV) are found in different tissues, where they present distinct structural and functional properties. Understanding the origin and characteristics of these collagen types is crucial for harnessing their potential in bioprinting and selecting the most suitable type and source. Type I collagen, predominantly found in the skin, tendons, and bones, provides structural integrity and support. It is commonly sourced from bovine and porcine tissues. Type II collagen is abundant in articular cartilage, where it contributes to its elasticity and strength. It is often derived from avian sources such as chicken sternum. Type III collagen is found in the skin, blood vessels, and internal organs and plays a crucial role in tissue elasticity. Bovine and porcine tissues are commonly used as sources for it. Type IV collagen is mainly present in basement membranes, representing an important fraction in the lung ECM, where it is involved in filtration and support. It is typically sourced from human placenta or recombinant technologies (Ricard-Blum, 2011).

The use of highly purified type I collagen derived from porcine sources has been undertaken for various applications. This versatile material can be 3D-printed directly or mixed with suspended cells to create cell-laden scaffolds. The inherent ECM composed of collagen contributes to the sustained viability of the encapsulated cells during extended periods of culture. Furthermore, using native biomaterials minimizes immune rejection upon transplantation, making them suitable for *in vivo* studies in the realm of TE.

Biomaterials sourced from marine environments present a compelling alternative to traditional mammalian-derived counterparts. This is primarily attributed to a reduced risk of disease transmission and greater compatibility with diverse religious and ethical beliefs in society. Type I collagen can be derived from a marine source (*Macruronus novaezelandiae*, blue grenadier). In a recent study, it was methacrylated to enable UV crosslinking during extrusion-based 3D printing (Maher et al., 2022). A recent study showed higher cytocompatibility of this material with L929 fibroblasts in comparison to the porcine-derived type I collagen; the mechanical properties of the marine-derived collagen, such as stiffness and tensile strength, generally exhibited lower values, indicating a softer and less rigid material than the porcine-derived one. The results showed that both types possess tunable Young’s modulus across a wide range. This study also concluded that despite its potential as a biomaterial in TE, the application of marine-derived collagen may be constrained by its lower thermal stability, leading to degradation into gelatin.

Fibrillar type I collagen from bovine sources has emerged as a standardized and reproducible collagen option for 3D printing and bioprinting applications. In a recent study, Fibercoll-Flex^N^ (Viscofan S.A., Tajonar, Spain) was used at 5% w/w and demonstrated exceptional performance during 3D printing, allowing for the generation of constructs with up to 27 layers without collapse. Furthermore, modifications to the fibrous collagen mass facilitated a rapid, dependable, and easily neutralizable process. The incorporation of cells was achieved through neutralization with Tris-HCl, balancing biocompatibility with printability. The resultant cell-laden constructs were successfully printed under mild conditions (50 kPa–80 kPa, pneumatic 3D printing), exhibiting remarkable cellular viability (>90%) and providing a stable platform for *in vitro* cell growth and proliferation (Garcia-Villen et al., 2023). Type I collagen from rat tail is also commercially available from different suppliers and is widely used as a scaffold material for 3D cell culture, nerve regeneration, and skin TE, among other applications (Xu et al., 2022).

Human skin collagen is currently under analysis for RM applications. HumaDerm and derivatives, commercially offered by Humabiologics, Inc. (Arizona, United States), are available in solution at concentrations of 3 mg/mL, 6 mg/mL, and 10 mg/mL. This product is designed for applications in cell culture and 3D hydrogel formulations (Bedell et al., 2023).

#### 2.1.3 Gelatin and GelMA

Gelatin is a denatured form of collagen traditionally used in pharmaceutical and biomedical applications (Wang et al., 2017). One of the most extensively researched gelatin-based biomaterials is gelatin methacrylate (GelMA), a chemically modified form of gelatin obtained through methacrylation, which enables photocrosslinking and tight control over the mechanical properties and stability of bioprinted structures.

GelMA stands out as a versatile biomaterial in bioprinting due to its ECM-derived biological cues, biocompatibility, and tunable viscoelastic and mechanical properties. These features make GelMA a preferred choice for formulating bioinks that closely mimic the physiological environment required for the optimal growth and functionality of encapsulated cells in TE applications (Bupphathong et al., 2022).

Several commercial GelMA formulations have been developed to standardize printability and mechanical tuning across different bioprinting platforms. GelMA INX R100 (BIO INX, Zwijnaarde, Belgium) and Claro™ BG800 (PB Leiner, Vilvoorde, Belgium) offer consistent and predictable rheological behavior suitable for extrusion-based bioprinting. Human-derived gelatin formulations, such as Huma OsteoGelatin (Humabiologics, Phoenix, United States), are obtained from AATB-certified human bone tissue and comply with FDA 21 CFR Part 1271. Their methacrylated derivative, Huma OsteoGelMA, provides photocrosslinking capability to further tune the mechanical properties for TE applications (Bedell et al., 2023).

#### 2.1.4 Hyaluronic acid (HA)

Hyaluronic acid (HA) is known for its capacities to retain water and promote cellular hydration, and it is used to improve the biocompatibility of bioinks. However, its low viscosity often necessitates blending with other polymers to achieve suitable printability and shape fidelity in 3D bioprinting applications (Can et al., 2020). To overcome this limitation, HA is frequently functionalized, for instance, through methacrylation, which enables photo-crosslinking and provides a method for tuning the mechanical properties of hydrogels (Rossi et al., 2024). Methacrylated hyaluronic acid-based bioinks are typically used at concentrations ranging from 1% to 4%, where higher concentrations enhance the structural stability and viscosity, while lower concentrations improve printability and cellular response (Huang et al., 2024).

#### 2.1.5 Chitosan

Chitosan, a linear polysaccharide derived from chitin, is increasingly being recognized for its distinct biological properties and structural versatility in TE, particularly in cartilage regeneration and wound healing. Its favorable biocompatibility, biodegradability, and antimicrobial characteristics make it an appealing component in various bioink formulations (Bill and Andrea, 2024). For instance, chitosan with glucomannan and glycerol phosphate salt, crosslinked with tripolyphosphate, has successfully been used in bioprinting applications for cartilage, skin, and bone (Agarwal et al., 2022). Furthermore, modified chitin and chitosan derivatives have demonstrated enhanced printability and mechanical properties, alongside the capacity to modulate immune responses and growth factor delivery within TE constructs (Turnbull et al., 2020).

#### 2.1.6 Nanocellulose (CNF/CNC)

Nanocellulose, a variety of the homopolysaccharide cellulose, is widely recognized for its versatile applications in bioprinting, and despite partially biodegrading, it has been developed as a biomaterial in the realm of TE and RM (Wang et al., 2020). Derived from plant-based sources, nanocellulose exhibits interesting properties, including high surface area, mechanical strength, and biocompatibility, making it an optimal candidate for bioink formulations. Several nanocellulose formulations with specific rheological characteristics were shown to be suitable components to formulate inks for 3D printing (Chinga-Carrasco, 2018). In fact, they provide a sustainable and renewable alternative in the field of bioprinting, aligning with the growing emphasis on eco-friendly approaches for biomedical research. Adding alginate to nanocellulose and cross-linking it with CaCl_2_ is a suitable approach to create porous structures using 3D printing (Espinosa et al., 2019).

### 2.2 Synthetic hydrogels and ready-to-use bioinks

Synthetic hydrogels such as polyacrylamide (Denisin and Pruitt, 2016; Norris et al., 2021), polyethylene glycol, PEG (Piluso et al., 2021), and acrylate-based polymers (Ding et al., 2019) play a crucial role in diverse applications ranging from contact lenses to controlled drug release. In addition, they are being analyzed as candidates for bioink formulations in various bioprinting technologies and applications.

Within this context, two widely used options are Stable INX R100® (BioINX, Zwijnaarde, Belgium) and VitroINK® (TheWell Bioscience, North Brunswick, United States), which stand out as ready-to-use xeno-free bioinks (Gebeyehu et al., 2021). VitroINKs® is available in both unmodified and modified forms, incorporating peptide motifs such as arginylglycylaspartic acid (RGD) or isoleucine–lysine–valine–alanine–valine (IKVAV) to enhance cellular functions including cell attachment and migration. Such advancements in bioink biomaterials technology opened up new possibilities in the field of TE, providing researchers and practitioners with versatile tools for creating biocompatible constructs with tailored properties.

Other compounds that play a fundamental role in bioink formulations or serve as integral components include HA (Antich et al., 2020), chitosan (Bagheri et al., 2019), chondroitin sulfate (Shin et al., 2021), and agarose (Zarrintaj et al., 2018). These materials not only add diversity to the available bioink options but also offer unique properties that enhance the ink’s abilities to replicate specific biological environments.

### 2.3 Synthetic polymers and thermoplastics

Due to their specific properties, thermoplastics are particularly valuable in the manufacture of the structural components of 3D scaffolds. These materials have the ability to be printed in uniform mesh-like structures, with good capacity for the maintenance of spatial geometry. They serve as a framework for the overall construct, enabling the stimulation of the construct with substantial mechanical loads, which would be impossible if only cell-laden hydrogels were used. Additionally, they play a significant role in the degradation rate of the construct (Oberdiek et al., 2021).

#### 2.3.1 Polycaprolactone and conventional polyesters

Biodegradable polymers can be broadly classified based on their origin (natural or synthetic) and their processing characteristics. Among synthetic biodegradable polymers, thermoplastics are a significant group widely used in TE. Many thermoplastic compositions have already received approval for clinical applications by the Food and Drug Administration (FDA). Their use as implants depends on the specific application, regulatory approval, and how the material has been processed or modified. One of the first studies on biodegradable thermoplastics used for implantation was presented in 1966 (Kulkarni, 1996), in which the biocompatibility of poly-L-lactic acid (PLLA) in animal models was studied. The material proved to be non-toxic and gradually degraded *in vivo*. Nowadays, materials such as polycaprolactones (PCLs) (Lopez-Gonzalez et al., 2021), polyhydroxyalkanoates (PHAs), and polydioxanones (PDO and PDS), along with poly(lactic acids) (PLAs), poly(glycolic acids) (PGAs), and their copolymers PLGAs, are used in biomedical applications (Kontakis et al., 2007).

#### 2.3.2 Flexible thermoplastic elastomers and conductive TPUs

Flexible thermoplastics that can be printed using fused deposition modeling (FDM) technologies are also gaining attention as their properties replicate the energy-absorbing behavior of joints. For example, 1,4-butanediol thermoplastic polyurethane elastomer (b-TPUe) has proven to be an interesting novel material for 3D-printing applications in the biomedical field (Chocarro-Wrona et al., 2021). In addition, electrically conductive TPUs have been specifically designed for the manufacture of scaffolds with applications in musculoskeletal and neural TE and for the creation of wearable devices, among other applications, since they allow for the transmission of mechanical and electrical stimuli.

#### 2.3.3 SEBS and other emerging thermoplastics

More recently, the SEBS (styrene–ethylene–butylene–styrene) family of thermoplastic elastomers was also analyzed for the fabrication of microfluidic devices and scaffolds for biomedical applications (Etayo-Escanilla et al., 2024). Optically transparent SEBS represent a great alternative to silicone polymers polydimethylsiloxane (PDMS) as they are certified for biocompatibility (ISO 10993 and USP Class VI), have higher hydrophilic stability, and are resistant to adsorption of small particles such as drugs or growth factors. In addition, SEBS can be printed directly from pellets using FDM, thus having a series of technical and economic advantages in comparison to their equivalents in the filament format (Banerjee et al., 2019).

#### 2.3.4 Blends and additive-reinforced thermoplastics

Blended thermoplastics and thermoplastics with additives show great potential for the short-term translation from the laboratory to clinical application. An example is PLA with osteoinductive particles such as magnesium or hydroxyapatite (Zhang H. Y. et al., 2020). For instance, Zhang *et al.* developed a bioresorbable magnesium-reinforced PLA membrane for guided bone/tissue regeneration. This composite, where a fluoride-coated magnesium alloy core is enveloped by PLA, demonstrated significantly enhanced mechanical properties (higher maximum load and stiffness) compared to pure PLA membranes. This design addresses the limitations of polymeric membranes in large bone defects by providing robust support. Additionally, the magnesium not only reinforces the structure but also influences the degradation of the PLA, with its degradation products potentially neutralizing acidic byproducts and promoting a favorable environment for bone formation.

Other thermoplastics used in TE are the family of polyethylene terephthalate glycol (PETG), which includes highly translucent copolyesters with high chemical resistance and durability. Polypropylene (PP) is a transparent, light, and flexible material with excellent mechanical properties and chemical resistance. Polyvinyl alcohol (PVA) is biodegradable, non-toxic, and water-soluble, and it was used in blends with other biomaterials such as chitosan to create support structures and achieve complex geometries with multi-extrusion printers (Chen et al., 2023).

### 2.4 Hybrid and composite biomaterials

Adapting the current manufacturing technologies or developing new ones for the production of novel scaffolds that support cells and the delivery of signaling molecules is necessary to improve the generation of *in vitro* tissue-like parts (McGivern et al., 2021). Using 3D bioprinting, strategies can be developed for creating multicomponent constructs composed of synthetic and natural polymers, taking advantage of the benefits of both types of materials (Baena et al., 2019).

Bioprinting allows us to manufacture multicomponent constructs composed of a scaffold that provides the support structure and a matrix of another biomaterial of hydrogel nature in which cells and other biomolecules are embedded. The matrix allows the cells to proliferate and differentiate in a biomimetic environment, while the supportive scaffold provides adequate conditions for stimulation (e.g., elasticity, high resistance to mechanical loads, and electrical conductance). The supportive scaffold (usually a thermoplastic) can be manufactured by technologies such as FDM, while ECM-derived biomaterials comprising the matrix where cells are embedded can be added using the “injection pore filling” (IPF) or “injection volume filling” (IVF) techniques in a multi-step bioprinting process (Baena et al., 2019; Antich et al., 2020).

### 2.5 Applications of specific biomaterials in cartilage regeneration

Natural hydrogels (alginate, agarose, fibrin, HA, gelatin, chitosan, chondroitin sulfate, collagen, and nanocellulose) have all been explored as bioactive scaffolds for cartilage regeneration (Li et al., 2019; Beketov et al., 2021). Synthetic polymers are currently being explored for cartilage repair, including polyesters, fumarates, and polyurethanes (Chung and Burdick, 2008), along with PLA and PGA (Cui et al., 2023), among others. Wu et al., 2021, in a systematic review, analyzed the potential of bioprinting of articular cartilage as a TE strategy with a tremendous capacity for clinical cartilage repair and regeneration, addressing damage caused by trauma or degenerative diseases, and future *in vivo* implantation (Wu et al., 2021). The Karami et al., 2023 review article provides a summary of the critical prerequisites that hydrogel-based devices should meet for cartilage repair, including successful implantation, preclinical validation, characterization techniques for the quantitative and qualitative outcome measures, and challenges in meeting the Good Laboratory Practice (GLP) guidelines (Karami et al., 2023).

### 2.6 Hydrogel limitations and advanced designs

Hydrogels typically lack the mechanical properties required to independently fulfill structural functions and are prone to swelling under physiological conditions, which can reduce the stiffness and compressive strength (Billiet et al., 2012). To address this limitation, Kamata et al. (2014) developed injectable “non-swellable” hydrogels from hydrophilic and thermo-responsive polymers, where two independently occurring effects (swelling and shrinking) oppose each other. The suppression of swelling helps retain the mechanical properties of hydrogels under physiological conditions. Subsequently, these hydrogels need to be tuned to be printable and cell-friendly (Kamata et al., 2014). An optimally hydrated environment is pivotal, not only for cell survival but also for the biofunctionality of the tissue. From the standpoint of 3D printing, hydrogels are strategically positioned to furnish and sustain such a conducive environment (O'Shea et al., 2022).

In the last few years, smart hydrogels with responsive properties to stimuli such as temperature, pH, or ion concentration have been developed. These biomaterials can change their properties based on environmental conditions (Arif et al., 2024). Particular attention was paid to the development of conductive hydrogels containing conductive particles such as carbon nanotubes or polymers (Distler and Boccaccini, 2020). Silk fibroin-gelatin with magnetic nanoparticles has also been analyzed with human bone marrow-derived mesenchymal stromal cells for cartilage regeneration (Chakraborty et al., 2024). Self-healing hydrogels are also gaining attention due to their ability to self-repair and regenerate after damage, making them suitable for long-lasting applications and smart-release drug-loaded hydrogels that enable controlled drug-release therapy (Caprioli et al., 2021).

ECM-derived hydrogels such as the previously mentioned Huma OsteoGelatin show huge potential in TE given that cells recognize them as a material found in native living tissues, thus increasing biocompatibility and integration. Other bio-inspired hydrogels are being explored, such as those containing the polysaccharides HA and alginate, for potential applications in 3D bioprinting of articular cartilage engineering constructs (Antich et al., 2020; Stone et al., 2023).

### 2.7 Scaffold architecture, CAD, and computational support

The scaffold architecture plays a major role in cellular behavior. Computer-aided design (CAD) libraries of scaffolds have been developed, and they represent useful tools for researchers who want to reproduce a specific structure. Different pore sizes and geometries can be tailored to develop structures with custom mechanical properties. These properties can be evaluated using finite element (FE) models (Chantarapanich et al., 2012). The advantage of AM fabrication is that it allows for the complex, hierarchical scaffold designs with different properties and architecture (Hutmacher et al., 2004; Tsang and Bhatia, 2004). The physicochemical features of a cell nanoenvironment may exert an important influence on cell behavior. The engineering of functional biomimetic scaffolds with programmed spatio-temporal physical and chemical signals for SC holds great promise in SC therapy (Das and Zouani, 2014).

Biomechanical characterization of hydrogels using computational models, such as finite element analysis (FEA), is necessary to better understand the processes behind design optimization and predict their behavior during bioprinting and post-implantation. Developing predictive computational models to forecast the effects of mechanical stimuli on constructs is key to guiding strategies that aim to produce functional implantable tissues *in vitro*.

### 2.8 Comparative summary table

Table 1 summarizes the biomaterials commonly used in cartilage tissue engineering and 3D bioprinting.

**TABLE 1:** Biomaterials for 3D bioprinting in cartilage tissue engineering.

Biomaterial	Key feature	Advantage	Limitation
Alginate	Natural polysaccharide; ionic crosslinking; high water content	Excellent printability; low cost; biocompatible; tunable viscosity; supports chondrogenesis	Low intrinsic bioactivity; weak mechanical properties; rapid degradation unless blended
Collagen (type I/II)	Major ECM component; fibrillar structure; strong cell-adhesive properties	Highly biomimetic; maintains chondrocyte phenotype; widely used in bioinks	Low mechanical strength; slow gelation; thermal instability
Gelatin/GelMA	Collagen-derived; thermoresponsive; photocrosslinkable	Good cell adhesion; tunable mechanics; excellent bioprintability; customizable crosslinking	Requires UV/photoinitiators; thermally sensitive; moderate mechanical robustness
Hyaluronic acid (HA)	ECM glycosaminoglycan; viscoelastic; hydrophilic	Promotes chondrogenesis; excellent biocompatibility; blends synergistically with other hydrogels	Very low mechanical strength; usually requires chemical modification for printability
Chitosan	Cationic polymer; forms stable hydrogels	Antimicrobial; biocompatible; adjustable degradation	Limited printability on its own; typically requires blending or chemical modification
Nanocellulose (CNF/CNC)	Fibrillar nanonetwork; strong rheological modifier; shear-thinning	High mechanical strength; excellent print fidelity; renewable source	Low bioactivity; usually combined with other biomaterials
PCL	Thermoplastic polyester; slow degradation; mechanically strong	Excellent structural support; ideal for FDM; enables load-bearing scaffolds	Hydrophobic; lacks bioactivity; very slow degradation
PLA/PLGA	Thermoplastics with tunable degradation	High mechanical strength; good dimensional accuracy; easy processing	Acidic degradation byproducts; limited elasticity
PCL + hydrogel hybrids	Multicomponent constructs combining stiffness and cell-compatible matrix	Balanced mechanical and biological performance; supports mechanostimulation	Multi-step fabrication; higher complexity
Composite hydrogels	Hybrid matrices combining natural and synthetic polymers	Improved printability; enhanced cellular response; customizable properties	Requires optimization; potential batch variability

## 3 Articular cartilage tissue and mechanobiology

### 3.1 Embryogenesis and cellular composition

Cartilage is a specialized connective tissue found in various parts of the body, such as joints, intervertebral discs, and the respiratory tract. Cartilage embryogenesis is the crucial process that involves the formation of cartilaginous tissue from precursor cells and is essential for the development of skeletal structures, including bones, joints, and respiratory structures (Hall and Miyake, 2000). At the gastrulation stage, the mesoderm matures into the mesenchyme, which is an undifferentiated type of connective tissue composed of precursor cells that have the potential to differentiate into various cell types, including chondroblasts (precursor cells) and chondrocytes (specialized cells), which are the main cell types making up cartilage tissue (Pacifici et al., 2006).

Chondroblasts are responsible for the production and secretion of the ECM of cartilage. These cells synthesize key components of the matrix, such as type II collagen, proteoglycans, and glycosaminoglycans (GAGs) (Archer and Francis-West, 2003). As cartilaginous tissue develops, chondroblasts mature into chondrocytes, the cell type found within lacunae in the cartilaginous matrix. Chondrocytes play an essential role in the maintenance and remodeling of cartilage during the development and adult life (Olsen et al., 2000).

Embryonic cartilage development is regulated by a combination of biochemical and mechanical cues. Growth factors such as transforming growth factor-beta (TGF-β) and fibroblast growth factor (FGF) orchestrate chondroblast proliferation and differentiation (Kronenberg, 2003). Mechanical forces generated during fetal movement, particularly compression and tension, further promote chondrogenic differentiation and contribute to physiological tissue maturation (Pacifici et al., 2006).

### 3.2 Historical foundations of cartilage histology

Histological studies of cartilage date back to the 17th century, when Marcello Malpighi first recognized cartilage as a distinct tissue with characteristic structural features. In the 19th century, Rudolf Virchow made major contributions to the field by identifying chondrocytes and describing an ECM rich in collagen and proteoglycans (Hunziker, 2002).

Throughout the 20th century, significant advances were made in understanding the histology of cartilage and its lesions. Staining and microscopy techniques have been developed to allow for a more detailed visualization of the structure of cartilage and its cellular and extracellular components. In addition, various growth factors (e.g., IGF-1, TGF-β, and FGF) and cytokines (e.g., IL-1 and TNF-α) involved in the regulation of metabolism and cartilage repair have been identified (Moo et al., 2001).

### 3.3 Extracellular matrix and zonal organization

The ECM of cartilage is mainly composed of type II collagen, proteoglycans, and water. This provides cartilage with its unique biomechanical characteristics, such as compressive strength and flexibility (Ateshian and Mow, 2005). There are three major cartilage types, namely, hyaline cartilage (the most abundant, found in synovial joints), elastic cartilage (e.g., pinna and epiglottis), and fibrocartilage (e.g., intervertebral discs and pubic symphysis) (Benjamin and Evans, 1990).

**FIGURE 2:**
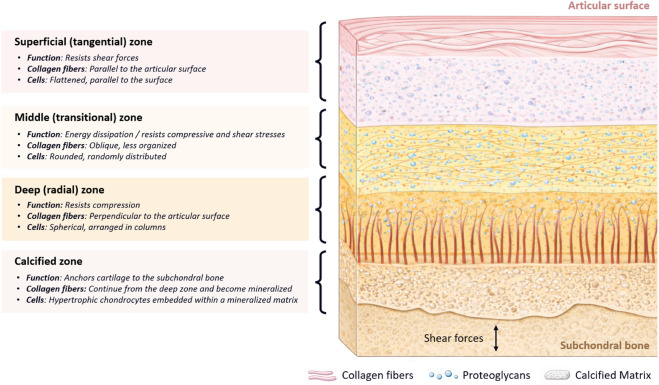
Schematic representation of the zonal organization of articular cartilage overlying the subchondral bone. The superficial, middle, deep, and calcified zones show distinct collagen fiber orientation, extracellular matrix composition, and cellular organization, contributing to depth-dependent mechanical functions such as shear resistance, energy dissipation, and compressive load transfer.

Articular cartilage exhibits a highly organized and heterogeneous depth-dependent structure that underlies its biomechanical function (Figure 2). This hierarchical architecture is reflected in distinct ECM organization, collagen fiber orientation, and cellular morphology across four main zones:

- Superficial (tangential) zone: collagen fibers aligned parallel to the surface, providing resistance to shear forces.
- Middle (transitional) zone: oblique and less organized collagen fibers with abundant proteoglycans, facilitating energy dissipation and load distribution.
- Deep (radial) zone: collagen fibers oriented perpendicular to the surface, anchoring cartilage to the subchondral bone and enhancing resistance to compressive loads.
- Calcified cartilage zone: mineralized region forming the interface between cartilage and subchondral bone.

As illustrated in Figure 2, this zonal organization establishes a functional structural gradient that enables efficient load distribution across the osteochondral unit. These depth-dependent structural and mechanical gradients are key regulators of cellular mechanotransduction and cartilage homeostasis (Ateshian and Mow, 2005; Athanasiou et al., 2015).

### 3.4 Biomechanical properties and loading regimes

The biomechanics of articular cartilage describe how the tissue responds to external forces and how these forces influence the physiological and pathological processes. Mechanical loading, deformation, loading speed, and loading duration impact the biomechanics of articular cartilage. The ability of cartilage to withstand, distribute, and recover from these forces is essential for joint function.

The behavior of articular cartilage is described by biomechanical properties, such as Young’s modulus, shear resistance, and damping capacity. Young’s modulus is a measure of the stiffness of a material, describing cartilage’s ability to resist deformation. Shear strength represents the potential to resist shear forces. Damping capacity refers to the ability of cartilage to absorb and dissipate energy during the loading cycle (Buckwalter and Mankin, 1998).

Importantly, articular cartilage is capable of adapting to the mechanical demands to which it is exposed. The load-dependence phenomenon describes how the biomechanical properties of cartilage can change in response to different loading regimes. Proper mechanical loading promotes ECM synthesis and chondrocyte function, leading to the formation of stronger and healthier cartilage. On the contrary, an excessive or unbalanced mechanical load can lead to degeneration and the appearance of joint diseases (Darling and Athanasiou, 2005).

### 3.5 Mechanotransduction mechanisms

When a load is applied to the tissue, it deforms and experiences stresses and strains that are proportional to the applied load. The tissue’s ability to resist and redistribute these stresses is critical for its function. Mechanical stimuli are thought to induce conformational changes in integrins, thereby regulating gene expression and tissue remodeling through the process of mechanotransduction (Millward-Sadler and Salter, 2004).

Mechanical forces are sensed by chondrocytes and mesenchymal stem cells through a coordinated network of mechanosensors, including integrins and focal adhesions, mechanosensitive ion channels such as PIEZO1/2 and TRPV4, the primary cilium, and the actin cytoskeleton. These structures convert external mechanical cues into intracellular biochemical signals that regulate chondrogenic behavior and cartilage homeostasis. This intricate process involves the activation or suppression of various intracellular signaling pathways following the stimulation of these mechanoreceptors (Gilbert et al., 2021).

Different types of mechanical stimuli activate distinct intracellular pathways. Dynamic compression typically activates TGF-β/Smad and MAPK pathways, increasing SOX9 activity and promoting type II collagen and aggrecan synthesis. Shear stress modulates Ca^2+^ flux through ion channels and stimulates lubricin (PRG4) expression. Tensile strain reorganizes the cytoskeleton and influences YAP/TAZ activity, thus affecting proliferation and phenotype maintenance. Hydrostatic pressure regulates Wnt/β-catenin signaling and supports chondrogenesis while limiting hypertrophic differentiation. Together, these mechanotransduction pathways enable chondrocytes to adapt to their mechanical environment and maintain cartilage homeostasis, directly influencing ECM deposition, cell survival, and tissue maturation (Dieterle et al., 2021).

Together, these mechanotransduction pathways enable chondrocytes to adapt to their mechanical environment and maintain cartilage homeostasis, thus directly influencing ECM deposition, cell survival, and tissue maturation. This complex interplay of mechanical cues and cellular responses is crucial for the continuous repair and regeneration of cartilage, preventing degenerative conditions such as osteoarthritis (Stanczak et al., 2025).

### 3.6 Mechanobiology in disease progression and *in vitro* models

Cartilage injuries can vary in their severity and clinical presentation. Among the most common injuries are cartilage degeneration and breakdown. Cartilage degeneration can be caused by factors such as age, acute trauma (e.g., sport injuries or car accidents), mechanical stress, joint disease, and metabolic disorders. These conditions can cause damage to either the cartilage ECM and/or chondroblastic cells, leading to the formation of free cartilage fragments in the joint, which cause pain and functional limitations. In severe cases, the injury can affect the underlying subchondral bone and cause osteochondral defects. Due to the avascular and aneural nature of articular cartilage, healing of these defects and regeneration of healthy tissue is highly limited (Krishnan and Grodzinsky, 2018).

Mechanobiology provides a framework to understand how mechanical forces influence these degenerative processes. It is an interdisciplinary field focused on how cells sense and respond to the mechanical properties of their microenvironment, integrating concepts from physics, biology, and engineering (Ingber, 2003; Wang and Thampatty, 2006; Wang et al., 2009). Recent decades have seen rapid growth in this area driven by advances in experimental platforms and imaging technologies—such as atomic force microscopy, cell traction systems, and optical manipulation—that allow detailed evaluation of cellular deformation and force transmission. Similarly, computational modeling and multiscale simulations have become essential for predicting the mechanical behavior of tissues and engineered constructs and for dissecting the signaling pathways that mediate responses to mechanical cues (Narasimhan et al., 2020).

This technological expansion has facilitated the development of diverse *in vitro* models. In a comprehensive review, Hodgkinson et al. (2022) summarized 2D and 3D systems—including single-cell, co-culture, and tissue-level constructs—used to analyze mechanobiology in cartilage and bone, particularly in the context of osteoarthritis onset and progression (Hodgkinson et al., 2022).

Comparing native cartilage mechanics with engineered biomaterials is critical for evaluating their suitability for regeneration. Weizel et al. (2020) compared the complex mechanical behavior of articular cartilage from three cadavers of 62-year-old women to commercial hydrogels used for cartilage repair (ChondroFiller liquid and ADA-GEL). The three types of samples were evaluated under recovery test, cyclic compression, cyclic tension, cyclic compression–tension, and other load combinations using a Discovery HR-3 Rheometer from TA instruments (New Castle, Delaware, United States). All materials exhibited nonlinearity and compression–tension asymmetry. However, while hyaline cartilage yields higher stresses in tension than in compression, the commercial hydrogels evaluated showed the opposite trend, concluding that these characteristics can be attributed to the materials’ underlying microstructure. Both cartilage and ChondroFiller liquid contain fibrillar components, but the latter constitutes a bi-phasic structure, where the 60% non-fibrillar hydrogel proportion dominates the mechanical response. Among all materials tested, ChondroFiller liquid showed the most pronounced viscous effects (Weizel et al., 2020).

These comparisons highlight a broader challenge: most *in vitro* loading paradigms oversimplify the complex and multiaxial mechanics present in joints such as the knee, where tissues experience concurrent compression, tension, shear, torsion, and hydrostatic pressure (Zhang L. et al., 2020). Reproducing this complexity is essential to guide physiologically relevant mechanotransduction.

Mechanical stimulation exerts strong and spatially dependent effects on ECM production. Early studies in the last century showed that cultured bovine articular cartilage subjected to dynamic compression *in vitro* increased the synthesis of PGs; however, this was limited to certain loading frequencies and pressures and occurred in specific areas under and around the loaded site (Parkkinen et al., 1992). Other studies using hydrostatic pressure described changes in the structural organization of stress fibers in articular cartilage chondrocytes, concluding that cytoskeletal changes may be an integral part of the response of chondrocytes to hydrostatic pressure (Parkkinen et al., 1995). More broadly, chondrocytes of the articular cartilage sense mechanical factors associated with joint loading and maintain the homeostasis of the ECM by regulating the metabolism of factors such as PGs and collagens (Jortikka et al., 2000).

### 3.7 Technological advances in mechanobiology

Mechanobiology has expanded rapidly in recent decades due to advances in experimental and computational techniques that enable the precise modeling, measurement, and manipulation of forces at the cellular and tissue levels. These tools have strengthened the conceptual and experimental framework needed to analyze how mechanical cues regulate biological processes (Jaalouk and Lammerding, 2009).

The field has deep historical roots. As early as 1880, Pierre Curie observed the piezoelectric effect, demonstrating that biological tissues can generate electrical signals in response to mechanical forces. Subsequent developments in microscopy and force-sensing techniques enabled direct visualization of cell and tissue deformation under load. Later, foundational work by researchers such as Julius Wolff and Stephen Hales established key principles relating mechanical loading to tissue structure and adaptation (Wall et al., 2018). Together, these developments laid the groundwork for modern mechanobiology.

### 3.8 Mechanical stimulation strategies in cartilage regeneration

Mechanobiology has proven to be a fascinating and promising field that bridges physics and biology to better understand the underlying mechanisms in biological systems. As our comprehension of how mechanical forces influence biological processes deepens and new standardized solutions to stimulate arise, new opportunities open up to apply this knowledge to fields such as RM, TE, and bio-inspired materials design. Mechanotransduction pathways relevant to chondrogenesis continue to be elucidated (Zhang et al., 2016). Several stimulation approaches have been explored in the context of cartilage regeneration.

Athanasiou et al. (2015) provided a comprehensive review of the biomechanical stimuli utilized in cartilage regeneration. The study highlighted several methods, including direct compression at varying frequencies and strain levels, dynamic loading of cell-seeded hydrogels, and the application of hydrostatic pressure on thermoplastics. These techniques have garnered significant attention in the field. However, it is important to recognize that excessive mechanical stimuli can be harmful to cells. For instance, while hydrostatic pressure can be beneficial, applying it beyond the physiological thresholds has been shown to reduce matrix production and increase inflammatory cytokines such as interleukin-6 and tumor necrosis factor. The review also explored combined strategies, including the integration of mechanical stimuli with growth factors, ion-channel modulation as a supplementary mechanostimulatory cue, and the use of enzymes such as chondroitinase-ABC (C-ABC) or lysyl oxidase (LOX), among others (Athanasiou et al., 2015).

## 4 Types of stimuli applied

Different types of stimuli have been tested to enhance the quality and efficiency of cartilage tissue formation from 3D cell-laden constructs. The microenvironment of cells plays a crucial role in processes such as differentiation and physiological function (Spanoudes et al., 2014). Notably, cells within biological structures are exposed to a range of extrinsic stimuli, including light, temperature, pressure, torsion, tension, and compression, all of which profoundly influence cellular behavior and characteristics. Consequently, these factors exert a considerable impact on the overall performance of the encompassing tissues and organs.

In the context of articular cartilage, mechanical loading stands out as the primary external stimulus influencing its homeostasis and operational efficacy (Quinn et al., 1998). This mechanical loading serves as a potent catalyst for the anabolic regulation of the ECM within the cartilage. As a result, scientific efforts have been directed toward the design and implementation of mechanical stimulation systems for *in vitro* engineering of articular cartilage (Li et al., 2017; Salinas et al., 2018). These systems involve the integration of basic mechanical components into simplified bioreactor setups used for cultivating animal cells in artificial environments. The overarching aim is to induce mechanical stress within tissue-engineered constructs. In several instances, these methodologies have proven effective in stimulating chondrogenesis at both the cellular and molecular levels.

Nonetheless, existing bioreactor designs fall short in reproducing the intricate anatomical configuration and subsequent physiological load distribution characteristic of human joints, which is a pivotal aspect for appropriate tissue maturation (Sanchez-Adams et al., 2014). Moreover, these systems are not specifically tailored to faithfully replicate the physiological motion shown by joints. Consequently, they run the risk of inducing excessive or non-physiological mechanical stimuli, which has been associated with articular cartilage degeneration and the onset of OA (Bader et al., 2011).

In the following section, the main types of stimuli—mechanical, biochemical, hydrodynamic/pressure-based, electrical/acoustic, and microgravity—are reviewed, along with their impact on chondrogenesis and ECM formation.

### 4.1 Mechanical stimulation

Mechanical stimulation is one of the key stimuli used to promote cartilage formation in 3D scaffolds. It consists in the application of biomechanical forces such as intermittent compression, uniaxial tension, cyclical deformation, and hydrodynamics. Such forces can promote the differentiation and function of cartilage precursor cells, leading to an increase in the synthesis of the main components of cartilage ECM (Bonassar et al., 2001; Thompson et al., 2016). Studies have reported significant improvements in the quality of cartilage tissue generated when mechanical stimulation is applied (Salinas et al., 2018).

Dynamic compression on chondrocyte-seeded peptide hydrogels and an alternate-day loading protocol significantly enhanced proteoglycan synthesis by up to two-fold higher than free-swelling cultures (Kisiday et al., 2004). Compressive loading of engineered tissues is often chondrogenic, increasing GAG synthesis and equilibrium modulus. Tensile loading is often fibrogenic, increasing collagen synthesis and tensile properties (Baker et al., 2011; Grad et al., 2011).

Other structures, such as the meniscus, may also affect the load distribution. Puetzer and Bonassar (2016) analyzed the effects of the load stimuli on this tissue. Axial loading (with tensile and compressive components) of anatomically shaped, tissue-engineered meniscus constructs produced spatial distributions of local strain similar to those seen in the meniscus when the knee is loaded at full extension. Such loading drove the formation of tissue with large and organized collagen fibers, levels of mechanical anisotropy and compressive moduli that match the native tissue (Puetzer and Bonassar, 2016).

Some multiaxial systems have been explored. Behrendt et al. (2020) created tissue constructs using human bone marrow-derived mesenchymal stromal/SC encapsulated in tyramine-modified hyaluronic acid (HA-Tyr) hydrogels. These were crosslinked by horseradish peroxidase (HRP) at various concentrations (0.3 mM–2 mM). The constructs were stimulated with a device providing multiaxial loading, consisting of 10% compression superimposed onto a 0.5 N preload and shear loading and applied at 1 Hz for 1 h per day, five times a week for 4 weeks, concluding that the applied stimuli activated endogenous TGF-β (Wimmer et al., 2004).

Shear stress is another important mechanical cue that acts coplanar with a given cross-section of the material and can influence mechanotransduction in cartilage tissue. Gemmiti and Guldberg (2009) demonstrated that this stimulus is a potent modulator of both the amount and type of synthesized ECM constituents in engineered cartilaginous tissue with corresponding effects on the mechanical function. The analyzed effects of shear stress on chondrocytes include the following: first, alterations in the expression of aggrecan and collagen type II; second, modifications in cartilage oligomeric matrix protein (COMP) serum levels, influencing the organization and binding of GAGs, integrins, and collagen; third, initiation of apoptotic signals; finally, alterations in the expression of integrins (Sharifi and Gharravi, 2019).

Altogether, mechanical loading remains one of the most powerful inducers of cartilage-like tissue formation. Nonetheless, most existing systems do not mimic the complex multiaxial loading of human joints, especially the knee.

### 4.2 Biochemical and nanoparticle-based stimulation

Biochemical stimulation plays a fundamental role in inducing SC differentiation into chondrocytes and promoting ECM synthesis. The combination of different biochemical factors and their controlled release in the scaffold have been shown to improve cell viability and cartilage formation (Somoza et al., 2014; Taheri et al., 2023). Growth factors such as TGF-β (Leipzig et al., 2006) and platelet-derived growth factor (PDGF), supplements such as ascorbic acid or insulin–transferrin–selenium (ITS), and inducers including dexamethasone constitute some examples.

As a part of biochemical stimulation, nanoparticles are also being studied to stimulate the generation of cartilage tissue as an effective way to deliver specific compounds locally or to apply magnetic fields or wireless technologies that can influence ECM formation (Bhattacharyya et al., 2022). Nanoparticles can be coupled with growth factors for remote activation by light or ultrasound. The combination of gold microchips and magnetic nanoparticles was shown to remotely monitor inflammation, mechanical/structural parameters, and the modulation of the ordered, spatial alignment of nano-biomaterials (Cafarelli et al., 2021).

### 4.3 Perfusion, hydrodynamic flow, and pressure-based stimuli

Perfusion bioreactors provide continuous medium flow through porous scaffolds, generating hydrodynamic shear stress while simultaneously improving nutrient supply and waste removal. This dynamic culture environment more closely mimics physiological tissue conditions than static culture, thus enhancing cell viability, promoting differentiation, and increasing ECM deposition. By ensuring homogeneous oxygen and nutrient delivery, perfusion also improves the distribution of cells throughout the scaffold and supports more uniform tissue formation.

The benefits of perfusion have been demonstrated in multiple studies. Chondrocytes seeded on PLLA/PGA scaffolds were analyzed under static culture and direct perfusion bioreactor conditions to assess the effects of fluid flow and media pH on matrix assembly. Results showed that these conditions significantly increased the DNA, GAG, and hydroxyproline content in the bioreactor compared to those in the static culture. This indicated the potential for rapid *in vitro* expansion of donor cartilage for various reconstructive surgeries and tissue repair applications (Pazzano et al., 2000). Similarly, 3D-printed PCL scaffolds incorporating adipose MSCs were cultured in both a static culture and a perfusion bioreactor. The findings revealed that dynamic culture conditions were more conducive to chondrogenic differentiation, promoting superior cell penetration throughout the scaffold compared to that in the static culture (Theodoridis et al., 2019).

Hydrostatic and osmotic pressure have also been analyzed to stimulate chondrocytes through short applications of high pressure to enhance Ca^2+^ concentration. In addition, changes in Na^+^ were reported in response to osmotic and hydrostatic pressure, with the effects of the latter on sulfate incorporation being influenced by extracellular osmolality, indicating location-dependent variations in chondrocyte responses within the joint (Browning et al., 2004). Hydrostatic pressure positively influences MSC chondrogenesis, enhancing the expression of genes and matrix proteins. However, there is an ongoing debate regarding the optimal timing for inducing the most beneficial response and whether it prevents hypertrophy. The underlying mechanisms of the hydrostatic pressure response require further exploration, potentially unveiling new therapeutic targets if novel pathways contributing to its positive effects are identified (Pattappa et al., 2019).

In a notable study, intermittent hydrostatic pressure (10 MPa, 1 Hz, 4 h/day, 5 days/week) applied to scaffold-free self-assembled chondrocyte constructs significantly increased collagen production and prevented GAG loss over 8 weeks of culture, demonstrating the strong chondrogenic potential of physiologically relevant pressure regimes (Hu and Athanasiou, 2006).

### 4.4 Electrical and acoustic stimulation

Electrical stimulation is another promising approach for generating cartilage in 3D scaffolds, as the application of electrical currents or fields can promote cell migration, ECM synthesis, reorientation, and cartilage tissue formation. Cartilage is a biphasic tissue: the solid phase is made of a charged collagen–PG matrix with large macromolecules with high polarity and negative charge (i.e., GAGs) and an interstitial fluid phase. It has varying biomechanical properties due to changes in its composition and different collagen fiber arrangement. Resistivity increases gradually from the superficial to deep zones, indicating a higher concentration of mobile charged particles in the superficial zone, which conducts electrical charge more efficiently. Elastic modulus increases and permeability decreases from the superficial to the deep zones (Vaiciuleviciute et al., 2023). The application of low-voltage and low-frequency electromagnetic fields showed positive effects on cell proliferation and differentiation and on ECM synthesis (Ciombor et al., 2002). Additionally, electrical stimulation can modulate the activity of ion channels and the expression of cartilage-related genes, leading to increased production of matrix and improved mechanical properties of the formed tissue. Electrical stimulation has been shown as a promising tool for enhancing articular cartilage TE outcomes by combining hydrogels such as HA or gelatin with MSCs or other biomaterials (Vaca-Gonzalez et al., 2020).

Acoustic stimulation, especially ultrasound, has also been widely explored to promote cartilage formation (Choi et al., 2013). Low-intensity ultrasound (LIUS) was shown to boost fracture healing in humans by increasing PG expression. Ultrasound prompts calcium influx in primary chondrocytes, indicating that calcium signaling mediates PG synthesis and can accelerate fracture repair (Parvizi et al., 2002).

Low-intensity pulsed ultrasound (LIPUS), an acoustic wave that produces mechanical stimulation over cells, is an FDA-approved technology for clinical therapy and bone fracture healing (Uddin et al., 2016). It was shown to promote the gene expression of type II collagen and enhance the synthesis of chondrogenic ECM (Martinez-Moreno et al., 2019).

Ultrasounds can also serve as non-invasive tools for the quality control of cell-laden scaffolds for cartilage regeneration by measuring the ECM formation. Melchor et al. identified a significant correlation between GAGs and type II collagen expression with the elastic damping evolution of *de novo* ECM. This finding reinforces the feasibility of using ultrasound to evaluate chondrocyte functionality and its potential to monitor chondrocyte proliferation and ECM formation in the context of 3D cartilage engineering. Ultrasounds can serve as a tool to evaluate the quality and functionality of the newly formed tissue, as it is an accurate and non-invasive procedure that can be automated. This will facilitate regulatory approval of bioprinted treatments and accelerate their translation from laboratory research to clinical application (Sebastian et al., 2023). Furthermore, the study concluded that ultrasounds can be used as a way of interacting with the biological components, producing local effects that may improve the quality of the regenerated tissue (Melchor et al., 2018).

### 4.5 Microgravity and multimodal strategies

Microgravity simulates the reduced mechanical environment of space, promoting cell aggregation (e.g., to study spheroids and organoids), and allows for the formation of 3D structures. Exposure to microgravity has strong effects on a variety of tissues, leading to decreased bone and muscle density and hematological alterations, among others (van den Nieuwenhof et al., 2024). One of the effects is the reduction in cartilage thickness due to a decline in GAGs (Ganse et al., 2022). However, recent evidence indicates sex-dependence in the transcriptomic response of bioengineered human cartilage to parabolic flight in microgravity. Specifically, upregulation of *WNT7B* and *WNT9A*, which are genes associated with osteoarthritis, is seen exclusively in female donors (Aissiou et al., 2023).

Despite these physiological challenges, researchers are analyzing the effects of microgravity on the generation of an environment that is similar to the one found during embryogenesis. Microgravity offers excellent advantages for TE by modulating cellular morphology, metabolism, secretion, proliferation, and SC differentiation (Cui et al., 2023). Early studies indicate its potential to improve our understanding of cartilage morphogenesis and inspire new regenerative strategies.

Beyond individual physical cues, combining multiple stimuli can better meet the biological requirements of complex and multi-material scaffolds. Hybrid stimulation approaches integrate mechanical, thermal, biochemical, or electromagnetic cues to enhance the formation and quality of engineered tissue. For example, Stampoultzis *et al.* analyzed the effect of loads and heating and found that the combination of dynamic thermal and mechanical stimuli induced superior effects in the expression of major chondrogenic genes, such as *SOX9* and *LOXL2*, compared to that with either signal alone. Similar synergistic effects were also observed in PG accumulation over time, together with increased mRNA transcription and synthesis of TRPV4 and, for the first time in chondrocytes, TREK1 ion channels. In contrast, the chondrogenic response of cells to isolated thermal or mechanical cues was generally dependent on the scaffold type. Nonetheless, the significance of thermal stimulation as a chondro-inductive signal was better supported in both the studied groups. They concluded that temperature evolution is necessary for chondrocytes to more effectively perceive and translate applied mechanical loading (Stampoultzis et al., 2023a).

Advances in medical imaging and computational processing—such as CT scanning, MRI, and high-resolution segmentation—combined with state-of-the-art 3D-printing technologies, now enable the faithful reconstruction of human joint anatomy (Mendonca et al., 2023). These anatomically accurate models can be seamlessly incorporated into next-generation bioreactor systems specifically designed for cartilage TE.

The integration of 3D anatomical models with programmable mechanical actuators allows precise replication of physiological joint movements within controlled culture environments. Such systems support the maturation of engineered cartilage by delivering joint-specific combinations of compression, rotation, shear, and multiaxial loading. In the final section of this review, we discuss several available platforms, including the BMAP® Knee bioreactor (REGEMAT 3D, Granada, Spain). The BMAP® system (where BMAP stands for *bioreactors that mimic the anatomy and physiology*) is designed to reproduce the anatomy and physiology of the knee, while simulating the movements that occur in the joint, including flexion–extension, uniaxial compression, rotation, and lateral displacement. Additionally, it controls and monitors the temperature, CO_2_ levels, and pH, while maintaining an aseptic environment. This paradigm shift also aligns with the evolution of personalized 3D bioprinting strategies, collectively enabling the fabrication of constructs that authentically mirror the comprehensive structural, mechanical, and biochemical complexity that is inherent to human articular cartilage.

### 4.6 Comparative summary table

Table 2 summarizes the main mechanobiological stimuli used in cartilage tissue engineering.

**TABLE 2:** Summary of common stimuli used in cartilage mechanobiology, their key characteristics, representative platforms, and design rationale.

Stimulus type	Key characteristics	Representative platforms	Design rationale
Compression (static/dynamic)	Uniaxial or multiaxial loading; regulates ECM synthesis; mimics physiological joint loading	Compression bioreactors; piston-driven systems	Enhances GAG and collagen II deposition; replicates cartilage’s load-bearing environment
Shear stress	Fluid-induced or surface shear; generated by movement or perfusion	Perfusion bioreactors; rotating wall vessels; custom shear chambers	Reflects lubrication/sliding forces in joints; promotes mechanotransduction
Hydrostatic pressure	Cyclic pressurization without deformation; characteristic of deep-zone cartilage	Hydrostatic pressure chambers; sealed pressurized bioreactors	Mimics intra-articular pressure; enhances chondrogenesis and ECM organization
Tensile loading	Stretching of scaffold or matrix; aligns fibers; relevant for superficial zone	Stretch bioreactors; flexible membrane systems	Stimulates collagen fiber alignment and zonal architecture
Chemical cues	Growth factors, cytokines, biochemical factors; exogenous media supplementation	Media supplementation; controlled-release scaffolds; nanoparticles	Drives chondrogenic differentiation (SOX9, COL2A1, and ACAN) and matrix remodeling
Electrical stimulation	Weak electric fields or pulsed currents; modulates ion flux and signaling pathways	Electrostimulation chambers; conductive scaffolds	Enhances ECM production and regulates chondrocyte phenotype

## 5 Computational simulation methods for articular cartilage

Computational methods for mechanical simulation have long supported clinical decision-making, implant design, and the optimization of regenerative strategies. These tools also enable the evaluation of physical therapies aimed at promoting cartilage formation (Huang et al., 2010). Historically, scientists have sought to understand the underlying mechanisms of the process. Recent advances in computational methods for mechanical simulation have enabled better understanding of tissue generation, particularly through the finite element method (FEM) (Clift, 1992).

The first attempts at computational simulation in mechanics date back to the 1950s, when numerical methods began to be developed to solve differential equations that describe the mechanical behavior of materials. The FEM was introduced in the 1960s and enabled the division of the domain of interest into discrete elements to solve equations within each element (Turner et al., 1956). Since then, this approach has become one of the most widely used methods in mechanical simulation due to its flexibility and ability to model complex geometries.

The primary aim of mathematical modeling and numerical simulation in TE is to predict the scaffold behavior and optimize structural design tailored to its intended function. This involves ensuring the appropriate pore size, interconnection, and permeability to facilitate cell movement and adhesion. In cartilage and bone TE, scaffolds must also possess sufficient strength to withstand mechanical loads from joints, which is determined by their macroscopic mechanical properties (Sanz-Herrera et al., 2008).

Computational models encompass a broad range of techniques—from FEM to molecular-scale simulations and artificial intelligence (AI)-based predictive systems—to analyze the tissue structure, elasticity, fluid flow, nutrient transport, and mechanical and biological responses. These multiscale approaches provide detailed insights into how tissues respond to different stimuli and conditions, which can be crucial for understanding diseases, designing therapies, and optimizing medical treatments. We can discover a diverse array of models that represent the crucial interactions among structural components, living entities, and other active biomolecules. These models are presented in various formats, including single- and multivariable equations, 2D/3D models, and even 4D models that include time as the fourth dimension. The literature offers diverse examples of specific tissue applications utilizing one or more of these computational models. Each presents its own advantages and limitations, and the choice of the most suitable approach depends on the specific research question and available data.

### 5.1 Continuum-level mechanical models: FEM and CFD

The FEM is the main model for analyzing mechanical distributions under different load conditions, such as by simulating bone mechanics to analyze stress distribution under different loading conditions. The FEM uses differential equations to describe the mechanical behavior of each finite element, and by combining these elements, a numerical representation of the system is obtained that can simulate and predict the mechanical behavior in response to different conditions and stimuli. In the FEM, various types of elements are utilized to simulate biological biomaterials. The common element types include the following:

- Solid elements for soft tissues and solid structures (muscles, tendons, skin, and cortical bone).
- Shell elements for thin biological membranes (pericardium and synovial membrane).
- Beam elements for thin elongated structures (blood vessels and tendons).
- Contact elements for interfaces (bone–bone and implant–tissue);
- Fluid elements for modeling fluid flows (synovial fluid in joints and blood in blood vessels).
- Interface elements for transitions between tissue types (e.g., ligament insertions) (Davol et al., 2008).

FEM has become indispensable in scaffold development, as demonstrated by Sanz-Herrera et al. (2009a), who used multiscale models to predict bone ingrowth as a function of scaffold porosity, stiffness, and degradation kinetics (Sanz-Herrera et al., 2009a; b). Similarly, Oberdiek et al. (2021) successfully modeled PCL and PCL + BCP scaffolds, predicting stress distribution and mechanical performance under compressive load—findings that aligned closely with experimental results (Oberdiek et al., 2021).

In cartilage TE, FEM enables the simulation of joint-level mechanics, predicting how compressive, tensile, and shear forces modulate ECM deposition, fluid pressurization, and cellular responses (Korhonen et al., 2013; Hassan et al., 2019). A notable example is the poroelastic FEM of the knee developed by Koh et al. (2019), incorporating patient-specific imaging. Their predictions demonstrated that optimal scaffold mechanical properties correlate with enhanced cartilage regeneration, highlighting the value of computational guidance prior to fabrication (Koh et al., 2019).

Complementary to FEM, computational fluid dynamics (CFD) models simulate fluid flow and associated phenomena using numerical methods and computer algorithms. These models solve the Navier–Stokes equations that describe the motion of viscous fluid substances to predict the behavior of fluids in complex geometries and under different boundary conditions. Widely used in engineering and biomedical applications, CFD models are utilized to study blood flow in arteries, airflow in the respiratory system, and drug transport in tissues, consequently aiding in the design of medical devices and therapies. Within the field of cartilage TE, CFD models are interesting tools for the evaluation of synovial fluid circulation in joints, leading to a better understanding of lubrication and tissue wear (Pekkan et al., 2003).

### 5.2 Microstructural and molecular simulation approaches: MD and LEMs

Molecular dynamics (MD) simulations model the movement of atoms and molecules over time. In such simulations, the interactions between atoms and molecules are described by potential energy functions, allowing researchers to study the dynamics of complex systems at the atomic level. MD simulations can provide insights into various phenomena (e.g., protein folding, molecular diffusion, and chemical reactions). They are widely utilized in the fields of chemistry, biophysics, materials science, and drug discovery to understand molecular-level processes and design novel materials and drugs. Simulating interactions between proteins and implant materials to predict biocompatibility is an interesting application of MD for TE (Chatterjee et al., 2021).

Lattice element models (LEMs) represent complex tissues such as cartilage as a network of interconnected nodes and springs, allowing for the simulation of their mechanical behavior. They can capture tissue microstructure, anisotropy, and heterogeneity. LEMs can reproduce compression, tension, and shear responses, aiding the understanding of joint function and degenerative processes. Although LEMs offer detailed insights, they may require significant computational resources and accurate parameterization for optimal performance (Sandino et al., 2010).

In 3D bioprinting, the optimization of bioink components and properties is crucial. For instance, polyacrylamide-alginate hydrogels, with their double-polymeric structure and high water content, are biphasic materials to which cells are added. This composition contributes to their complex mechanical behavior, making computational models valuable tools for evaluating and predicting their properties. However, assessing the fracture resistance of these hydrogels presents significant challenges. Current approaches often involve applying methodologies designed for hard solids to gels with extreme softness and high deformability. These include non-linear fracture mechanics for highly stretchable polyacrylamide gels, peel-test-like methods to evaluate the fracture energy correlated with cross-linking density and crack speed, and the use of linear elastic fracture mechanics (LEFM) to derive fracture energy from the stress intensity factor under mode I (KI) based on their proposed elastic system models (Reinhards-Hervás et al., 2024). Hydrogels also have a high swelling ratio with large deformation that can increase up to 30 times their original volume when exposed to aqueous solutions. A biphasic continuum-level swelling model utilizing the mixed hybrid FEM (MHFEM) in 3D has been proposed to accurately capture this procedure and enhance the accuracy of calculating the dynamics of swelling (Yu et al., 2018).

The mechanical behavior of gel-like tissues is intricately linked to mechanotransduction phenomena. Fennell and Huyghe (2021) explored the influence of ionic concentration on the modulus of hydrated tissues and hydrogels and proposed the modeling of the mechano-electrochemical relationship of hydrogels to quantify the coupling of elastic and electrochemical energies. By adapting the Flory–Rehner theory and incorporating material-specific experimental data, the study revealed differences in equilibrium swelling magnitude compared to the original model. Furthermore, it observed an increase in electrical potential with heightened shear strain under isochoric deformation, a phenomenon overlooked in conventional theories. This closer alignment between the continuum model and experimental observations indicated potential applications in both biological and technological contexts, particularly where mechanosensing capabilities and variable swelling responses are of interest (Fennell and Huyghe, 2021).

### 5.3 Cell-scale and tissue-scale predictive models

Cell mechanics models simulate the mechanical behavior of individual cells, considering factors such as cell shape, cytoskeletal structure, and interactions with the ECM. These models often utilize principles from continuum mechanics and biomechanics to predict cell deformation, migration, and response to mechanical stimuli. They play a crucial role in understanding complex processes such as cell adhesion, division, and differentiation, thus representing a valuable tool in biomedical research and TE applications (Haider et al., 2010).

A cellular automaton is a discrete computational model consisting of a grid of cells, each of which can exist in a finite number of states. Cells interact with each other based on a set of predefined rules, which is typically determined by the state of a cell and the neighboring ones. The state of each cell is updated synchronously in discrete time steps according to these rules, leading to emergent patterns and behaviors at a global level, such as tissue organization or population dynamics. Cellular automata have been widely used to model complex systems and phenomena in various fields, including physics, biology, computer science, and artificial life (Wurthner et al., 2000). Cellular automata were proposed to simulate cell dynamics, aiming to enhance outcomes in cell culture procedures by assessing crucial factors such as migration, proliferation, and cell death. This approach, which has been applied to different cell types, underscores the importance of experimental validation. To address this, a cellular automata model grounded in random-walk theory was proposed to forecast the articular chondrocyte behavior during monolayer culture cell expansion. The results indicated that the model adeptly mirrors cell dynamics, offering accurate quantitative insights (Vaca-Gonzalez et al., 2017).

Agent-based simulation models represent complex systems by simulating individual entities, or agents, and their interactions in a dynamic environment. These models capture behaviors that arise from interactions between agents, allowing researchers to study complex phenomena such as crowd behavior, spread of diseases, and ecosystem dynamics. Agent-based models are highly adaptable and scalable, making them valuable tools in various fields including epidemiology, ecology, sociology, and economics for scenario analysis and policy evaluation. One application in TE could be the study of cellular population dynamics in tissue formation during embryonic development (Christley et al., 2007).

Simulation of articular cartilage generation using computational methods has provided valuable insight into the biological processes and biomechanical interactions involved in cartilage development and maintenance. These simulations have allowed us to analyze how mechanical factors (e.g., load and deformation) influence the differentiation and proliferation of cartilage cells, along with the production and distribution of the components of the ECM (Korhonen et al., 2013; Hassan et al., 2019). Sophisticated computational tools are indispensable to further address the intricate details of cartilage physiology and pathology and analyze the various mechanisms at play. Among these, computational methods of mechanical simulation, particularly the FEM, offer a valuable tool to analyze and understand the processes involved in the generation of articular cartilage. These simulations provide detailed information about biomechanical interactions and factors influencing cartilage formation and function, which may have important implications for the development of RM treatments for joint diseases (Puetzer and Bonassar, 2016). The process to be simulated in the specific case of cartilage remodeling is the following: mechanical forces are believed to cause changes in the shape of integrins, which in turn influence gene expression and tissue remodeling through the process known as mechanotransduction. This is the mechanism by which cells convert mechanical stimuli into biochemical signals, leading to various cellular responses. Integrins are proteins found on the surface of cells, where they act as receptors that guide cell attachment to their surrounding ECM. When mechanical forces are applied, integrins can change their shape or conformation and consequently send signals into the intracellular space. This can affect the activity of various genes and modify the type and quantity of proteins being expressed. As a result, cells can alter their behavior, including growth, differentiation, and movement. This contributes to the restructuring or remodeling of tissues (Millward-Sadler and Salter, 2004).

Another work proposed a 3D FE model enhancing a previous mathematical formulation (Cortez et al., 2016) by integrating two anisotropic approaches to simulate the transport of nutrients, cell growth kinetics, ECM synthesis, and remodeling of biphasic mechanical properties within a hydrogel. FE also introduced a novel remodeling algorithm aimed at predicting the arcade-like collagen structure within a layered PCL scaffold for cartilage TE. The presented FE model offered a numerical analysis of collagen fiber evolution and orientation across the three zones of a layered PCL scaffold. This computational tool can aid in better comprehending experimental tests in cartilage TE (Cortez et al., 2017).

### 5.4 AI-driven optimization and machine learning models

Artificial intelligence approaches are becoming central to computational biofabrication. ANN models are also gaining attention within the scientific community. ANN is a computational model inspired by the structure and function of biological neural networks in the human brain (Rosenblatt, 1958). It consists of interconnected nodes, or “neurons,” organized in layers. Each neuron receives input signals, processes them through an activation function, and generates an output signal. ANNs are capable of learning from the generated data by adjusting the strength of connections between neurons through a process known as training. They are used in various applications such as pattern recognition, classification, prediction, and optimization tasks. One example of the use of ANN in cartilage TE is predicting cellular response to different growth factors in tissue regeneration (Khalvandi et al., 2022). Their ability to incorporate non-linear biological behavior makes ANNs valuable for predicting emergent tissue-level outcomes from cell-scale data.

Cartilage TE can also benefit from disruptive machine learning technologies. On one hand, we can work on improving bioprinting processes by optimizing parameters; on the other hand, we can attempt to predict the complexity of biological systems. Biofabrication processes pose significant challenges due to their complexity and often yield unsatisfactory results. Empiric improvements can be slow and costly, while providing partial results. Traditional trial-and-error methods are time-consuming and typically lead to minor improvements, starting from suboptimal existing processes. Computational techniques offer a promising avenue for more efficient process design by identifying optimal configurations. However, to be effective, these computational approaches must incorporate the complexity inherent in biological systems to provide meaningful insights (Shin et al., 2022).

In recent studies, a novel co-simulation-based optimization methodology was proposed for systematically designing protocols for cell culture and biofabrication. This approach integrates evolutionary computation and simulation techniques to explore the design space efficiently and evaluate candidate protocols. A generic library supports the modular and flexible composition of multiscale and multi-domain co-simulation scenarios. The feasibility of this approach was demonstrated through the automatic generation of protocols for biofabricating an epithelial cell monolayer. First, the prototyped co-simulation library facilitated the creation of flexible and loosely coupled simulation scenarios; second, the *in silico* experimentation showed that the proposed approach represents a viable initial step toward standardizing and automating the design processes in biofabrication (Giannantoni et al., 2022). Reinforcement learning-based computational design space exploration methodology has also been used to generate optimal protocols for simulated fabrication, such as in the creation of epithelial sheets. The optimization strategy relies on a deep reinforcement learning algorithm, the Advantage Actor–Critic, which is based on a neural network model for learning. In contrast, simulations depend on the PalaCell2D simulation framework (Castrignanò et al., 2024).

Despite rapid progress, most computational methods still focus on isolated stages of the biofabrication process. The need for integrated multistage models that combine biological, mechanical, and technological aspects still remains (Bardini and Di Carlo, 2024).

### 5.5 Applications for biofabrication, bioreactor design, and multiscale challenges

Computational modeling is also a useful tool for the evaluation of the performance of bioreactors for 3D cell cultures’ stimulation. For example, using computational models, we can increase our understanding of shear stresses arising from bioreactor fluid flow, optimize scaffold designs, tailor the mechanical characteristics of engineered cartilage tissue, and simulate cell growth rates and dynamics. Insights gained from virtual studies can effectively augment both *in vitro* and *in vivo* research endeavors. Nonetheless, it is crucial to acknowledge and rationalize the constraints of such models, including the assumptions and simplifications inherent in each one. Moreover, efforts should be made to validate and verify *in silico* models that can integrate the use of more traditional and known approaches (Pearce et al., 2020).

From an engineering standpoint, scaffolds are multicomponent systems with a mesh structure that can be modeled as a rigid or elastic element that will degrade over time. In addition, the matrix presents viscoelastic and anisotropic properties and performance; it contains cells and other biomolecules that in some configurations can vary their mechanical behavior. Computational methods can help researchers predict the effect of load transmission through the scaffold based on the type of material and its geometry. The presence of structural variability, a defining characteristic of biological systems, extends across scales ranging from molecular to tissue levels and plays a crucial role in the functionality of cartilage. Articular cartilage shows a stratified arrangement caused by the non-uniform organization of its components, encompassing unevenly dispersed fluids, electrolytes, collagen fibers, PGs, and chondrocytes. The exceptional macroscopic mechanical properties of cartilage stem from the diversity observed among these layers, coupled with the intricate micro-mechanical behavior arising from the interactions among the constituents within each layer. Currently, there is a lack of comprehensive models that encompass the various phases and scales pertinent to the mechanics and mechanobiology of cartilage. This scarcity presents a significant opening for the development of upcoming models that can integrate patient-specific and extensive deformation mechanics with biological and biochemical aspects in 3D. Such models hold the potential to advance personalized treatments and the creation of soft tissue substitutes in the field of TE (Wang et al., 2019).

The examples presented here illustrate the diversity and relevance of computational tools for understanding the biological and structural processes. The next section will focus on the physical replication of these processes using devices that mechanically stimulate bioprinted constructs.

## 6 Current devices and future trends

The *ex vivo* creation of personalized cartilage tissues is a promising approach for transforming the treatment of joint disorders. However, the high cost associated with the production and the inconsistent results of tissue-engineered implants continue to hinder their widespread adoption. To enhance clinical efficacy and broaden the use of engineered tissues, there is a need for automated bioreactor systems that are capable of supporting and monitoring tissue development. A bioreactor provides a biologically active and controlled environment that critically influences the final properties of the cultured cells or engineered tissues. This environment directly affects cell viability, cell distribution within 3D scaffolds, and the preservation of cellular phenotypes and tissue characteristics (Freed and Vunjak-Novakovic, 1995; Langer et al., 1995). Moreover, bioreactors must administer physical stimulation *ex vivo* to growing tissues to promote ECM secretion and tissue formation (Martin et al., 2004), which may be applied in chemical, electrical, mechanical, or magnetic forms (Fu et al., 2021). Although the selection of appropriate biomaterials and bioprinting strategies is essential, the subsequent maturation of engineered tissues inside a bioreactor is equally—if not more—critical and challenging.

Current technologies still face important practical limitations. Many prototypes rely on repurposed equipment not originally designed for cartilage TE, and these systems often fail to reproduce the complex anatomical arrangement and physiological load distribution of human joints. As a result, they may apply unnatural or degenerative mechanical forces. Additionally, a lack of customization to accurately replicate natural joint motion can produce excessive or abnormal stimuli associated with articular cartilage degeneration and osteoarthritis (Fu et al., 2021). Overcoming these technological gaps is, therefore, essential for producing clinically relevant and reproducible implants.

To address these limitations and support robust cartilage formation *in vitro*, bioreactors must fulfill several key requirements. A sterile environment is essential to prevent contamination and maintain the viability and integrity of cell cultures. Precise monitoring and control of pH, nutrient flow, O_2_/CO_2_ concentrations, fluid dynamics, and pressure are required to support cell survival, promote differentiation, and recreate physiological or pathological microenvironments. Controlled mechanical loads can promote the differentiation and physiological function of chondrocytes, whereas imbalanced loading can be used to model pathological conditions such as osteoarthritis or chondromalacia. Additionally, the incorporation of biochemical signaling gradients that mimic *in vivo* zonation is essential to support the formation of engineered tissues with native-like structural and functional properties.

Beyond commercially available systems, additional insight into emerging strategies can be obtained from patented bioreactor designs. Patent WO2004046304A1 describes a hybrid bioreactor that applies compressive strain for cell differentiation and shear strain for cell proliferation simultaneously to cells. This hybrid bioreactor consists of reactor tube assemblies, a compressive strain motor, a shear strain motor, a lower anchor mount containing toothed anchors to anchor the lower ends of the reactor tube assemblies, a ball screw operated alongside the compressive strain motor, an upper anchor mount engaged with the ball screw to move vertically, equipped with compressive strain anchors to anchor the upper ends of the reactor tube assemblies, and a power transmission unit to transfer the rotational force of the shear strain motor to the toothed anchors (https://patents.google.com/patent/WO2004046304A1/en). Patent US9410113B2 outlines a bioreactor for 3D tissue stimulation composed of one or more growth chambers containing a fluid media reservoir and a construct positioned within a pressurized cavity. A control system regulates the pressure and temperature of the delivered gas, thus enabling defined mechanical cues to be applied to the tissue construct (patents.google.com/patent/US9410113B2/en).

In parallel with mechanical and environmental control, microscale and nanoscale features of the bioreactor–scaffold interface also plays a critical role in regulating cell behavior. Cells sense and adapt to surface topography through a multiscale cascade linking integrin-based adhesion to cytoskeletal organization and nuclear mechanotransduction. Nano- and microscale features modulate integrin clustering and focal adhesion maturation, thereby shaping actin dynamics, contractility, and downstream mechanosensitive pathways. Recent studies highlight that specific nanotopographical cues—including ligand nanospacing and curvature—can destabilize integrin clusters and reduce actomyosin traction while simultaneously activating non-canonical mechanosensing routes. In particular, large-spacing adhesive patterns promote rapid actin turnover and the nuclear import of G-actin, where its polymerization increases nuclear tension and remodels chromatin to regulate lineage-specific transcription (Liu et al., 2025). Complementary findings show that oriented or flexible fibrous topographies can generate distinct adhesion profiles and cytoskeletal architectures that influence MSC commitment and matrix production (Dong et al., 2024). These topography-dependent mechanisms are, therefore, essential when designing scaffold microstructures and bioreactor interfaces for cartilage TE, as they directly shape chondrogenic gene expression, ECM deposition, and the establishment of zonal organization in engineered constructs.

Considering these mechanobiological requirements, a wide variety of bioreactors has been developed for cell culture and TE applications. These include conventional spinner flasks (Freed et al., 1993), rotating wall vessels (Freed and Vunjak-Novakovic, 1995), concentric cylinder systems (Saini and Wick, 2003), perfusion bioreactors (Carver and Heath, 1999), and stirred-tank bioreactors used for large-scale cell production. While some bioreactors target large-scale cell expansion or the production of cell-derived products (e.g., vaccines), others are specifically engineered to maintain, mature, and mechanically stimulate engineered tissues. To improve clarity and conceptual integration, in this section, we group the different bioreactor systems according to their primary mechanical mode of stimulation and include simplified conceptual illustrations that summarize how each type of bioreactor applies mechanical cues and interacts with the cultured constructs.

### 6.1 Rotational and fluid-flow bioreactors

Rotational and fluid-flow bioreactors primarily expose constructs to controlled shear stress generated by fluid motion or rotational dynamics. In these systems, differences in maturation parameters strongly influence the structure and properties of developing constructs (Vunjak-Novakovic et al., 1996). Depending on the scaffold characteristics and cell type, an appropriate bioreactor configuration must be selected.

**FIGURE 3:**
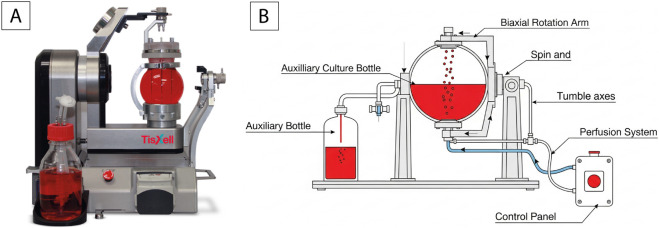
**(A)** TisXell bioreactor with biaxial rotation for 3D cell culture and tissue engineering. The system provides controlled environmental conditions and gentle biaxial motion to enhance nutrient and gas exchange and support mechanotransduction during construct maturation. **(B)** Schematic representation of the TisXell bioreactor showing the biaxial rotation arm, spin and tumble axes, auxiliary culture bottle, perfusion circuit, and control panel.

A prominent rotational device in this category is the TisXell bioreactor (I&L Biosystems GmbH), which utilizes biaxial rotation for TE applications (Figure 3). This system enhances nutrient and gas exchange through biaxial rotation and enables continuous soft irrigation by laminar flow, combined with additional mechanical stimulation via mechanotransduction. It has also been used in cartilage regeneration studies within a porous polysaccharide scaffold (Luciani et al., 2016). This was achieved through the synergistic combination of efficient magnetic condensation of mesenchymal stem cells and dynamic maturation processes executed within a TisXell bioreactor environment. Under optimal circumstances, all distinctive features of chondrogenesis exhibited remarkable enhancement. Type II collagen expression increased by a remarkable 50-fold when compared to that of a negative control, alongside elevated levels of aggrecan and collagen XI expression. Conversely, the expression of type I collagen and RUNX2 remained notably suppressed. Histological staining showed a substantial presence of cellular aggregates, accompanied by an increased synthesis of PGs by chondrocytes. Electron microscopy highlighted enlarged chondrocytes and heightened ECM deposition. Furthermore, the newly synthesized collagen fibers displayed a periodicity that closely matched that of type II collagen.

Fluid-flow systems have also been adapted for patient-specific anatomy. Lin et al. (2009) developed a scaffold–bioreactor system for tracheal growth and analyzed the effect of fluid flow on tracheal-like neo-tissue production (Lin et al., 2009). The Taylor–Couette flow-based bioreactor consists of a viscous fluid circulating in the space between two rotating cylinders. Zhu et al. (2010) reported the use of this device to grow bone marrow stromal (BMS) cell-sponge constructs. The results indicated that the stresses (0.02 Pa–0.19 Pa) generated in the bioreactor enhanced the BMS cell proliferation by approximately 1.3 times compared to the static control with a well-maintained calcium deposition capacity in the cells. Slightly higher shear stresses (0.24 Pa) promoted calcium deposition but inhibited proliferation, and the highest shear stress tested (40.24 Pa) inhibited proliferation altogether and impaired calcium ion retention (Zhu et al., 2010).

### 6.2 Compression-based bioreactors

Mechanical compression is one of the most physiologically relevant stimuli for articular cartilage. Several systems apply cyclic compressive loading that activates mechanotransduction pathways related to TGF-β signaling, collagen type II synthesis, and proteoglycan accumulation. Bioreactors such as the EBERS Medical TC-3F, Flexcell FX-5000™, and CellScale MCTX provide controlled compressive or tensile stimulation to 3D constructs.

The TC-3F by EBERS Medical Technology S.L. (Zaragoza, Spain) is an updated version of the TC-3 bioreactor. It applies forces to samples while allowing the user to monitor and measure tension or compression levels. Up to three experiments can be run simultaneously, while the force exerted by the material is recorded. This represents the first load system, which is specifically designed for cell culture with force measurement capabilities. To assess the impact of physiological deformational loading on cartilage tissue engineered constructs, an experiment was conducted, where PGA scaffolds were dynamically seeded with human chondrocytes and cultured under mechanical stimulation. Intermittent and unconfined direct compression was applied for up to 3 weeks, alongside a control group subjected to continuous low-speed perfusion. Both groups were maintained under standard culture conditions. After harvest, the constructs underwent biochemical, histological, and mechanical analyses. Results showed that the loaded constructs exhibited rich deposition of GAG with cells in lacunae, indicating improved cartilage production compared to that in unloaded controls. GAG and type II collagen fractions were significantly enhanced, with equilibrium moduli increasing from 52 kPa to 194 kPa in loaded constructs.

The Flexcell FX-5000™ Compression System (Flexcell®, Burlington, N.C.) is a computer-regulated bioreactor that applies cyclic or static strain to cells cultured on a pneumatically deformed membrane. These systems allow controlled mechanical loading of *in vitro* cell models, creating an environment that mimics *in vivo* mechanical conditions. Supported by a full range of TE accessories, these instruments facilitate the fabrication of 3D cell-seeded tissue constructs of varying shapes and sizes. Flexible 6-well and 24-well culture plates with different matrix coatings are also available to enhance the growth of diverse cell types. Due to their versatility, Flexcell® systems are widely used across biomedical research.

The CellScale MCTX (MechanoCulture TX), developed by Cellscale (Waterloo, Canada), is a commercially available platform capable of high-throughput uniaxial compression stimulation. It simultaneously compresses 3D specimens in six independent wells. User-defined loading protocols can be executed by the system, and the resultant force–displacement data are recorded to analyze the stiffness profile of each specimen over time. Transparent culture wells enable visual confirmation of correct specimen loading, and real-time imaging during testing is possible, when required. The specimen chamber plate is sterilizable, and the system is suitable for long-term cell culture within a laboratory incubator.

Several other companies offer bioreactors that are designed to deliver mechanical stimulation to cell cultures and biomaterials. Examples include Bose ElectroForce® (New Castle, Del.), BISS Tissue Growth Technologies (Bangalore, India), and UStretch® (Waterloo, ON, Canada). TA ElectroForce® BioDynamic® test instruments integrate bioreactor chambers with mechanical testing platforms to offer stimulation, characterization, and tissue growth solutions for engineered tissues and biomaterials in a sterile cell-culture media environment. UStretch® systems allow vertical and horizontal testing capabilities, in and out of a temperature-controlled media bath, with various specimen attachments such as screw-driven clamps, spring-loaded clamps, and multi-point puncture grips.

Despite offering controlled mechanical stimulation, these systems typically hold insufficient media volume to sustain cells for extended periods and pose challenges for medium exchange and optical monitoring during dynamic compression. Furthermore, the clamps used in most systems are limited in their application for soft hydrogels. These bioreactors are generally only suitable for monolayer cultures, and their capacity for applying relevant mechanical loads to developing tissues in a 3D context is limited. Therefore, there is a recognized need for the development of advanced research platforms that provide reliable, tissue-mimetic, and mechanically active 3D microenvironments.

McDermott et al. (2021) addressed some of these limitations by developing a custom bioreactor to culture hMSCs in fibrin hydrogels under varying durations of chondrogenic priming (0, 2, 4, or 6 weeks) before exposing them to static culture or dynamic compression for 2 weeks. They assessed the construct’s mechanical properties, cartilage matrix composition, and gene expression. The results indicated that dynamic compression increased the equilibrium and dynamic modulus of the engineered tissue, with the effect depending on the duration of chondrogenic priming. For priming durations of 2 weeks or more, dynamic compression enhanced the expression of COL2A1 and Aggrecan mRNA at the end of the loading period but did not affect total collagen or GAG matrix deposition. Initiating loading at priming durations of 4 weeks or less repressed transient osteogenic signaling (RUNX2 and OPN) and CYR61 expression, which is a YAP/TAZ-TEAD-target gene. However, no suppression of osteogenic gene expression was observed if loading commenced after 6 weeks of *in vitro* priming, during which mechanical stimulation increased the expression of type X collagen. Overall, the findings indicated that the duration of *in vitro* chondrogenic priming influenced the cellular response to dynamic mechanical compression, indicating that early loading may preserve chondrocyte homeostasis, while delayed loading may support cartilage maturation (McDermott et al., 2021).

Additional work demonstrated the effectiveness of compression in decellularized cartilage ECM-based constructs. An *ad hoc* bioreactor applying cyclic compression (10% strain, 1 Hz) for 2 weeks (3 h/day) to chondrocyte-laden dECM constructs—with or without IGF-1 supplementation—showed preserved cell viability and proliferation, increased expression of chondrogenic markers (COL2A1, ACAN, PRG-4), reduced MMP-3 expression, enhanced GAG content, and improved fiber alignment, leading to higher Young’s modulus (Sani et al., 2022). IGF-1 offered modest benefits but was less effective than mechanical loading alone; combined stimulation produced a synergistic increase in COL2A1 expression. Overall, mechanical loading applied to moldable cartilage dECM supports the formation of functional cartilage-like tissue.

Another study emphasized the role of synovium-derived cells in replicating the biomechanical microenvironment of the knee. They demonstrated that the early steps required for such a process can occur *in vitro*. First, they showed that under physiological mechanical loads, chondrocyte death occurred in the superficial cartilage zone alongside surface topographical changes. Second, synoviocytes were released from the synovial lining under physiological loads and attached to worn cartilage. Third, synoviocytes parachuted onto a simulated or native cartilage surface will modify their behavior. Specifically, they showed that synoviocyte interactions with chondrocytes lead to changes in synoviocyte mechanosensitivity and demonstrated that cartilage-attached synoviocytes can express COL2A1, which is a hallmark of the chondrogenic phenotype. Their findings indicated that synoviocyte-mediated repair of cartilage ‘wear and tear’ as a component of joint homeostasis is feasible *in vitro* and represents a promising area for further study (Pellicore et al., 2023).

### 6.3 Biaxial and multiaxial loading bioreactors

Complex bioreactors aim to reproduce combined biomechanical signals such as multidirectional compression, shear, torsion, and sliding articulation.

**FIGURE 4:**
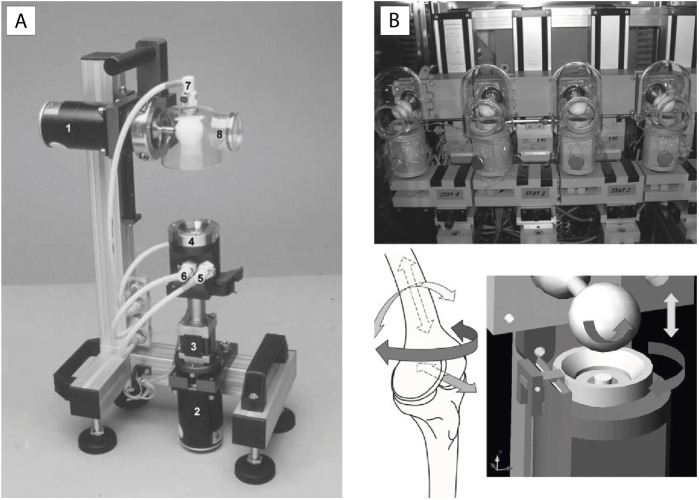
**(A)** Pin-on-ball bioreactor from Wimmer et al.’s work from 2004. **(B)** Four-station bioreactor capable of applying multiaxial load (Grad et al., 2011).

Loebel et al. (2017) developed tissue constructs using human bone marrow-derived mesenchymal stem/stromal cells enclosed in HA hydrogels modified with tyramine (HA-Tyr). These hydrogels were cross-linked by introducing the peroxidase enzyme from horseradish (HRP) along with varying concentrations of hydrogen peroxide (0.3 mM–2 mM). Constructs were then subjected to multiaxial loading, involving a 10% compression combined with a 0.5 N preload, followed by cyclic loading (625 cycles) at a frequency of 1 Hz for 1 h per day, 5 days per week, over 4 weeks. The study concluded that this applied mechanical stimulation activated the body’s internal transforming growth factor-beta (TGF-β) (Loebel et al., 2017). The apparatus used to apply these stimuli is detailed in Wimmer *et al.*'s work from 2004, the so-called pin-on-ball bioreactor, using a tribology apparatus for TE and the study of articular cartilage (Figure 4).

**FIGURE 5:**
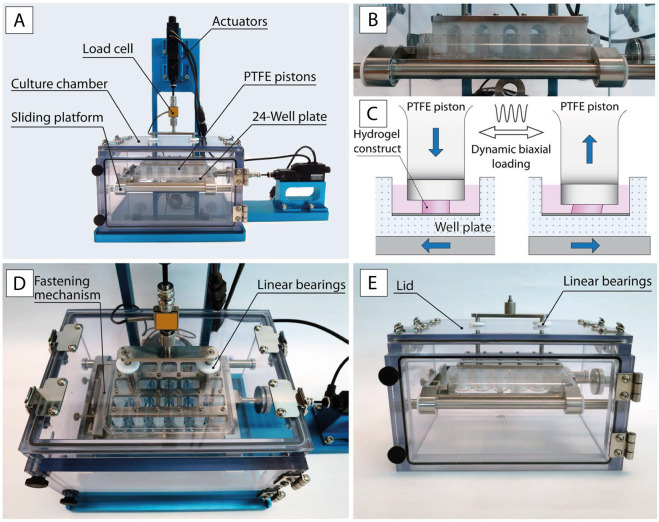
A novel bioreactor system for biaxial mechanical loading enhances the properties of tissue-engineered human cartilage (Meinert et al., 2017). **(A)** Overview of the bioreactor system. **(B)** Close-up view of PTFE pistons inserted into a standard 24-well plate. **(C)** Schematic representation of dynamic biaxial loading applied to hydrogel constructs. **(D)** Top view of the loading platform and fastening system. **(E)** Frontal view of the bioreactor chamber and linear bearing system.

Similarly, Ladner et al. (2023) and Meinert et al. (2017) developed multi-well or biaxial-load systems demonstrating strong upregulation of chondrogenic markers and enhanced ECM deposition. Ladner et al. (2023) introduced a new, simple-to-build and operate, multi-well, kinematic load bioreactor and analyzed its effect on the chondrogenic differentiation of human bone marrow-derived mesenchymal stem cells (MSCs). MSCs seeded into fibrin–polyurethane scaffolds were exposed to combined compression and shear forces for 25 days. Mechanical stimulation activated TGF-β1, upregulated chondrogenic genes, and increased sulfated GAG retention within the scaffolds (Ladner et al., 2023). Meinert et al. engineered a tightly controlled and automated system capable of applying precise uni- or biaxial mechanical stimulation for developing cartilage neotissues (Figure 5). The authors stated that the bioreactor allowed for simple control over the loading parameters with a user-friendly graphical interface and was equipped with a load cell for monitoring tissue maturation. They demonstrated that human articular chondrocytes encapsulated in hydrogels composed of GelMA and HA methacrylate (HAMA) responded to uni- and biaxial mechanical stimulation by upregulation of hyaline cartilage-specific marker genes. In addition, intermittent biaxial mechanostimulation enhanced the accumulation of hyaline cartilage-specific ECM. They underlined the stimulatory effects of mechanical loading on the biosynthetic activity of human chondrocytes in engineered constructs and the need for easy-to-use automated bioreactor systems in cartilage TE (Meinert et al., 2017).

Other researchers designed and validated an *in vitro* mechanical system capable of applying controlled bi-axial loading regimes to agarose constructs seeded with chondrocytes (Di Federico et al., 2014). Their computer-controlled system featured a robust gripping mechanism to ensure precise delivery of cyclic compressive and shear strain to the 3D cell-seeded constructs. They developed and optimized sample prototypes using FEA, followed by validation through compressive and shear fatigue mechanical tests. The system allowed for precise control of horizontal and vertical displacements within the bioreactor *via* a dedicated program that was easily implementable. Furthermore, they successfully loaded constructs with a combined compressive and shear loading regimen at 1 Hz for up to 48 h without significant loss of cell viability or mechanical integrity. The system’s features, combined with its demonstrated high consistency, make it suited for systematically analyzing the response of chondrocytes to complex and physiologically relevant deformation profiles.

**FIGURE 6:**
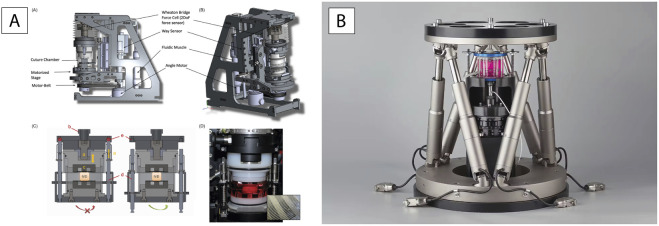
**(A)** Two-DoF IVD bioreactor. Reproduced from Gantenbein et al. (2019). **(B)** Six-DoF IVD bioreactor by CSEM.

While these systems focus on construct-level loading, more complex platforms have been developed to reproduce physiological motions at the organ scale. A first example of an intervertebral disc-specific loading device is the 2-degrees-of-freedom (2-DoF) bioreactor described by Gantenbein et al. (2019), which enables combined axial compression and torsional loading under controlled culture conditions (Figure 6A) (Gantenbein et al., 2019). Building on this work, the Swiss Center for Electronics and Microtechnology (CSEM) developed a 6-DoF spine motion platform capable of applying controlled translations and rotations in all anatomical axes, thus more accurately reproducing the complex multiaxial loading experienced by the human spine (Figure 6B).

In parallel, Secerovic et al. (2022) designed a 6-DoF IVD organ model and a customized specimen holder engineered to interface with an emerging multiaxial bioreactor system. The organ model maintained high cell viability after 3 weeks of cyclic compression in a uniaxial bioreactor and remained viable under physiological loading in the prototype multiaxial platform. The holder–IVD interface demonstrated mechanical stability under compression, torsion, bending, and tension, supporting its suitability for next-generation multiaxial IVD bioreactors (Secerovic et al., 2022).

**FIGURE 7:**
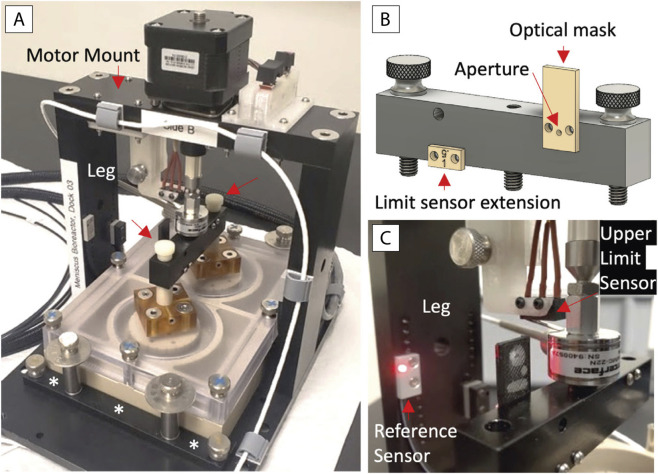
Development of a bioreactor for in vitro compression cycling of tissue-engineered meniscal implants. **(A)** General view of the dock showing the motor mount, support legs, piston tie-bar, and mounted culture chamber secured with removable bumpers and thumbscrews (asterisks indicate the front bumper). **(B)** Detailed view of the piston tie-bar including the optical mask aperture for reference sensing and the extension used to define the lower travel limit. **(C)** Side view of the sensor configuration showing the reference sensor and the upper limit sensor that prevents excessive actuator displacement. (Loverde et al., 2023).

Meniscal TE has also benefited from multiaxial platforms such as the modular compression bioreactor by Loverde et al. (2023), which is designed to reproduce anatomical force magnitudes and loading rates relevant to the knee (Figure 7). This modular system consists of a sterilizable culture vessel paired with a mechanical dock that applies and measures compressive forces. The vessel supports simultaneous cyclic compression of two anatomically sized menisci. A hybrid linear actuator capable of delivering forces up to 300 N at speeds of 20 mm/s provides physiologically relevant dynamic loading. An interchangeable 22 N load cell records force changes. Both the vessel and dock operate within a standard incubator to maintain the temperature and CO_2_ levels, while external software controls system operation (Loverde et al., 2023).

### 6.4 Perfusion and environment-controlled bioreactors

Perfusion bioreactors induce direct medium flow through the scaffold, improving oxygen and nutrient delivery and enhancing waste removal, thereby mitigating the mass-transport limitations associated with static culture. Several commercial platforms and integrated systems are used for environmental control and scale-up (Eppendorf BioFlo®, Sartorius BIOSTAT®, Thermo Fisher HyPerforma®, Pall Allegro™ STR, New Brunswick™ CelliGen®, Xcellerex™ XDR, PBS Biotech®, and CellMaker™); these platforms are important for process control and expansion, although they are not intrinsically designed to impose complex mechanobiological stimuli on tissue constructs.

Specialized perfusion platforms that combine interstitial flow with controlled biochemical environments have been developed specifically to promote MSC chondrogenic differentiation. For example, BIOMIMESYS® CARTILAGE (HCS Pharma) utilizes ECM-based hydrogels within a perfusion chamber to support 3D culture under controlled mechanical cues (Maubon, 2023). The CART-I® system—developed in the EU Horizon 2020 project “Bio-scaffold with *in situ* Cartilage Regeneration and Immuno-Modulation” (Grant Agreement ID: 646059)—provides precise oxygen and nutrient control to drive MSC differentiation toward the cartilage lineage, with the goal of producing *ex vivo* cartilage suitable for implantation.

Perfusion strategies have also been adopted to mimic patient-specific anatomy, as demonstrated in a temporomandibular joint construct. The authors reported that by the fifth week of cultivation, significant tissue growth was observed, including the formation of dense layers of lamellar bone, increased volume of mineralized matrix, and the presence of osteoids. Additionally, the density and structure of the bone matrix correlated with the intensity and pattern of interstitial flow, as determined through experimental and modeling studies (Grayson et al., 2010). These results are being used by EpiBone, Inc. (New York, United States), a privately held clinical-stage RM company focused on skeletal reconstruction.

Beyond perfusion, additional environmental modulators—including hydrostatic pressure (HP), oxygen tension (hypoxia), thermal cycling, ultrasound, and electrical stimulation—are increasingly being integrated into multimodal platforms. For example, Chariyev-Prinz et al. (2023) demonstrated density-dependent HP responses in hMSCs. They reported that high cell densities (15 × 10^6^ cells/mL) were more supportive of GAGs deposition (per cell), while collagen deposition was higher at lower seeding densities (5 × 10^6^ cells/mL) (Chariyev-Prinz et al., 2023). Utilization of hydrogels with lower fibrin (2.5%) concentration supported more robust chondrogenesis of hMSCs, with higher type II collagen and lower type X collagen deposition compared to that with 5% hydrogels. Their results demonstrated that the application of HP to hMSCs maintained in identified chondro-inductive culture conditions had little effect on the overall levels of cartilage-specific matrix production. However, if hMSCs were first temporally primed with TGF-β3 before its withdrawal, they responded to HP with increased GAGs production. This supports the importance of the ingredients used beyond the applied mechanical stimulus. Furthermore, they found that the response to HP in cultures with higher cell density was also associated with a metabolic shift toward glycolysis, which has been linked with a mature chondrocyte-like phenotype. These findings indicate that mechanical stimulation may not always be required to engineer functional articular cartilage grafts if other culture conditions are optimized, but HP-based systems remain valuable for studying mechanobiological responses once the tissues are removed from static conditions.

In another study, Stampoultzis et al. (2023b) showed that combining hypoxia with thermal–mechanical cues enhances chondrocyte mechanoresponsiveness and matrix formation (Stampoultzis et al., 2023b). Their three-module system integrates a PID-controlled thermal unit, a gas mixer regulating CO_2_ and O_2_ (normoxia or hypoxia), and a bioreactor chamber coupled to an Instron E3000 device to apply compressive loading. This multimodal stimulation significantly upregulated key chondrogenic genes.

Ultrasound has also been explored as a non-mechanical environmental stimulus to promote cell proliferation, matrix deposition, and tissue formation. Hsu et al. (2006) used pulsed ultrasound (1 MHz, 67 mW/cm^2^ Ispta and 10 min/day), allowing comparisons with the use of a rotating bioreactor (Hsu et al., 2006). The scaffolds were made of polyester composites and were fed with human chondrocytes obtained from the knee joint cartilage of three patients. The study concluded that ultrasound had a beneficial effect on neocartilage formation in the human chondrocytes-seeded, type II collagen-modified polyester composite scaffolds. Nevertheless, this effect was shown to decay after 28 days of culture, while that of bioreactors could last up to 42 days.

### 6.5 Anatomical and joint-mimetic bioreactors: toward personalized mechanobiology

Despite significant progress, most existing systems still fail to reproduce the complex anatomical architecture and physiological load distribution of human joints. Developing effective strategies for articular cartilage regeneration, therefore, requires advanced bioreactor platforms specifically designed for this purpose. Equally important is the design of 3D constructs—whether bioprinted or manufactured using other TE technologies—that reproduce the macroscopic organization of the tissue while enabling multi-scale mechanical stimulation.

Advancements in computational medical imaging, such as computed tomography and MRI, along with progress in 3D printing technology, offer a new opportunity to create anatomical and human-like mechanical stimuli. This involves creating precise 3D models that can be integrated into bioreactors designed for cartilage TE (Mendonca et al., 2023). By combining these detailed 3D models with mechanical actuators that mimic natural joint movements within a controlled environment, it becomes possible to replicate the complex anatomical and physiological conditions of human joints. These advancements will support the development of engineered cartilage constructs and align with the advancement of dedicated 3D bioprinting techniques. These collectively have the potential to construct tissue replicas that accurately capture the extensive structural, mechanical, and biochemical intricacies that are characteristic of human articular cartilage (Baena et al., 2019).

**FIGURE 8:**
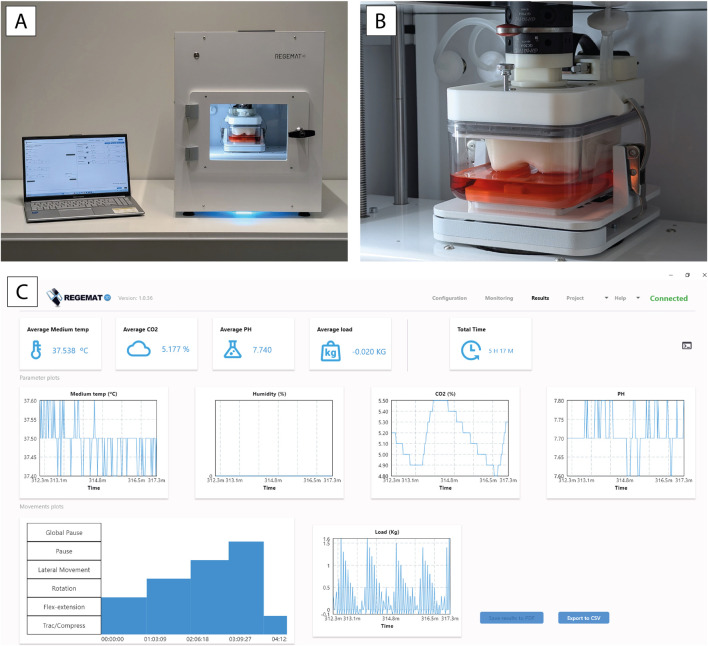
**(A)** Photograph of the BMAP® Knee bioreactor. **(B)** Photograph of the biocapsule containing culture media, showing the femoral condyles (distal femur) and the tibial plateau (proximal tibia). **(C)** BMAP® Knee software interface, where parameters such as the average medium temperature, CO_2_ levels, pH, and load can be monitored in real-time.

Recent developments by REGEMAT 3D (Granada, Spain) have led to the creation of the BMAP® Knee system, a bioreactor designed to reproduce patient-specific knee anatomy while maintaining the key physiological conditions—temperature, CO_2_, and pH—required for cartilage development. As illustrated in Figure 8, the system delivers controlled joint-like mechanical loading across the femoral–tibial interface within a sealed culture chamber, enabling the maturation of constructs generated through 3D bioprinting or other TE approaches and supporting the development of functional cartilage models.

To provide a structured overview of the currently available technological platforms, the representative commercial and research bioreactor systems and their main characteristics are summarized in Table 3.

**TABLE 3:** Overview of representative commercial and research bioreactor platforms used for mechanical stimulation in cartilage tissue engineering and mechanobiology studies.

Platform type	Representative system	Main mechanical stimulus	Physiological relevance level
Membrane deformation systems	Flexcell® platforms	Tensile strain/compression/hydrostatic pressure	Cellular and scaffold-level mechanobiology
Hydrostatic pressure systems	TissueGrowth technologies bioreactors	Hydrostatic compression	Chondrogenic differentiation and matrix synthesis
Dynamic compression platforms	Bose ElectroForce® bioreactors	Cyclic compressive loading	Mechanical conditioning of cartilage constructs
Advanced modular loading systems	TisXell bioreactors	Programmable compression and combined loading	Osteochondral tissue engineering and preclinical mechanobiology
Joint-mimetic/tribological simulators	Multiaxial joint simulators	Combined compression, shear and sliding	Tissue- and joint-level mechanical stimulation
Whole-joint anatomical bioreactors	BMAP® knee bioreactor	Multidirectional joint loading reproducing knee kinematics	Integrated joint-scale mechanobiology and cartilage regeneration

This overview highlights the technological evolution toward platforms that are capable of reproducing increasingly complex and physiologically relevant mechanical environments. These technological advances set the stage for the next-generation of mechanobiology-driven regenerative strategies.

### 6.6 Future perspectives

Although considerable progress has been made, substantial work is still required to fully leverage mechanically driven tissue and organ formation using next-generation bioreactor systems. The convergence of biomechanics, biofabrication, and advanced culture technologies indicates that the field is entering a transformative phase. Integrating anatomy-based mechanical loading, multimodal stimulation, and personalized scaffold design will likely define the next technological landscape in RM.

Ultimately, the development of anatomically accurate and multiscale mechanostimulation platforms will be essential to translate cartilage TE strategies from experimental settings toward clinically relevant regenerative therapies.
